# Haplotype-resolved genome assembly of the diploid *Rosa chinensis* provides insight into the mechanisms underlying key ornamental traits

**DOI:** 10.1186/s43897-024-00088-1

**Published:** 2024-04-16

**Authors:** Xiaoni Zhang, Quanshu Wu, Lan Lan, Dan Peng, Huilin Guan, Kaiqing Luo, Manzhu Bao, Mohammed Bendahmane, Xiaopeng Fu, Zhiqiang Wu

**Affiliations:** 1 National Key Laboratory for Germplasm Innovation & Utilization of Horticultural Crops, College of Horticulture and Forestry Sciences, Huazhong Agricultural University, Wuhan, 430070 China; 2 Shenzhen Branch, Guangdong Laboratory of Lingnan Modern Agriculture, Key Laboratory of Synthetic Biology, Laboratory of the Ministry of Agriculture and Rural Affairs, Agricultural Genomics Institute at Shenzhen, Chinese Academy of Agricultural Sciences, Shenzhen, 518120 China; 3 Kunpeng Institute of Modern Agriculture at Foshan, Foshan, 528200 China; 4 College of Science, Health, Engineering and Education, Murdoch University, 90 South Street, Murdoch, WA 6150 Australia; 5 Laboratoire Reproduction Et Development Des Plantes, INRA-CNRS-Lyon1-ENS, Ecole Normale Supérieure de Lyon, 520074 Lyon, France

**Keywords:** *Rosa chinensis*, Haplotype-resolved genome, Petal colour, Flower development

## Abstract

Roses are consistently ranked at the forefront in cut flower production. Increasing demands of market and changing climate conditions have resulted in the need to further improve the diversity and quality of traits. However, frequent hybridization leads to highly heterozygous nature, including the allelic variants. Therefore, the absence of comprehensive genomic information leads to them making it challenging to molecular breeding. Here, two haplotype-resolved chromosome genomes for *Rosa chinensis* ‘Chilong Hanzhu’ (2*n* = 14) which is high heterozygous diploid old Chinese rose are generated. An amount of genetic variation (1,605,616 SNPs, 209,575 indels) is identified. 13,971 allelic genes show differential expression patterns between two haplotypes. Importantly, these differences hold valuable insights into regulatory mechanisms of traits. *RcMYB114b* can influence cyanidin-3-glucoside accumulation and the allelic variation in its promoter leads to differences in promoter activity, which as a factor control petal color. Moreover, gene family expansion may contribute to the abundance of terpenes in floral scents. Additionally, *RcANT1*, *RcDA1*, *RcAG1* and *RcSVP1* genes are involved in regulation of petal number and size under heat stress treatment. This study provides a foundation for molecular breeding to improve important characteristics of roses.

**Supplementary Information:**

The online version contains supplementary material available at 10.1186/s43897-024-00088-1.

## Core

Two haplotype-resolved chromosome genomes for highly heterozygous* Rosa chinensis* were first reported. *RcMYB114b* can function as an activator of anthocyanin biosynthesis in petals. The regulatory mechanisms of flower development under high temperature were investigated.

## Gene and Accession Numbers

Sequence data and rose genome with annotation from this article can be found in the database of the National Center for Biotechnology Information (NCBI) under the accession numbers PRJNA932466.

## Introduction

Roses are the most popular cut flowers in the world due to their beautiful colours and rich fragrances. The cultivation of roses can be dated back 5,000 years among civilizations in Asia (Wang 2007). Notably, *Rosa chinensis* cultivars like ‘Old Blush’ (OB) and ‘Crimson China’ were highly significant old Chinese rose in the history of rose breeding that were introduced to Europe in the eighteenth century, and a large number of modern rose varieties were cultivated through frequent crosses of these cultivars (Huylenbroeck, 2018). Then, rose production entered a period of vigorous development, during which many of the most sought-after traits were cultivated (Young et al. 2007). Roses become the dominant species in cut flower production globally, owing to its appealing petal colours, intricate floral patterns, rich fragrances and high vase life.

To meet huge market demands and expedite breeding progress, many efforts have been invested in unraveling the molecular mechanisms underlying key traits. For petal colour, the genes involved in the anthocyanin biosynthesis pathway, *dihydroflavonol-4-reductase* (*DFR*) and *flavonol synthase* (*FLS*) (Suzuki et al. 2014), *MYB*, *basic helix-loop-helix* (*bHLH*) and *WD40*, were reported (Koes et al. 2005, Bendahmane et al. 2013, Raymond et al. 2018). For floral morphology, the differential expression of *AGAMOUS* (*RhAG*) in roses contributed to double-petal or simple-petal flower formation (Dubois et al. 2010), similar to findings in other species (Jung et al. 2014, Wollmann et al. 2010). Moreover, a microRNA172 (miR172) target-deficient *APETALA2-like* (*AP2L*) gene was correlated with the double-flower phenotype in rose (François et al. 2018), peach (Gattolin et al. 2018), and carnation (Wang et al. 2020, Zhang et al. 2022). For scent, different rose varieties exhibit different principal aroma components (Scalliet et al. 2008, Magnard et al. 2015, Zhou et al. 2020a). Additionally, with the ongoing change in global temperatures, and especially the frequent occurrence of high temperature (Seneviratne et al., 2021), it has a significant impact on flower yields and morphology in roses. The mechanism behind of these phenotypes are poorly deeply understood.

An increasing number of studies have provided preliminary evidence that allelic variations may play important roles during evolution and trait selection. In rose, the presence or absence of a retrotransposon insertion in the *Koushin* (*RcKSN*) allele determined the recurrent/once blooming character (Iwata et al. 2012). The duplication and specialization of *Nudix hydrolase 1* (*NUDX1*) in Rosaceae led to geraniol production in petals (Conart et al. 2022). In *Citrus*, one allele of *AbRuby2*^*Full*^ acted as an anthocyanin activator, while other *CgRuby2*^*Short*^ as repressor (Huang et al. 2018). Most of the ornamental plants are highly heterozygous, especially the rose. However comprehensive genomic information at a haplotype-resolved level is still lacking for the published rose genome (Chen et al. 2021, Raymond et al. 2018, Zhong et al. 2021).

Here, we present a high-quality, haplotype-resolved genome of *R. chinensis* ‘Chilong Hanzhu’ (‘CH’), a variety of the ‘Crimson China’ cultivar, which is one of the two significant old Chinese rose cultivars (in addition to ‘OB’). We identified a lot of variations and observed that the allelic genes exhibited differential expression between two haplotypes. We performed the regulatory mechanisms involved in important ornamental traits, and identified key related regulatory genes. Our findings provide fundamental resources and insights for accelerating the genetic improvement of roses.

## Results

### Assembly and annotation of the haplotype genome of R. chinensis ‘Chilong Hanzhu’

**Fig. 1:**
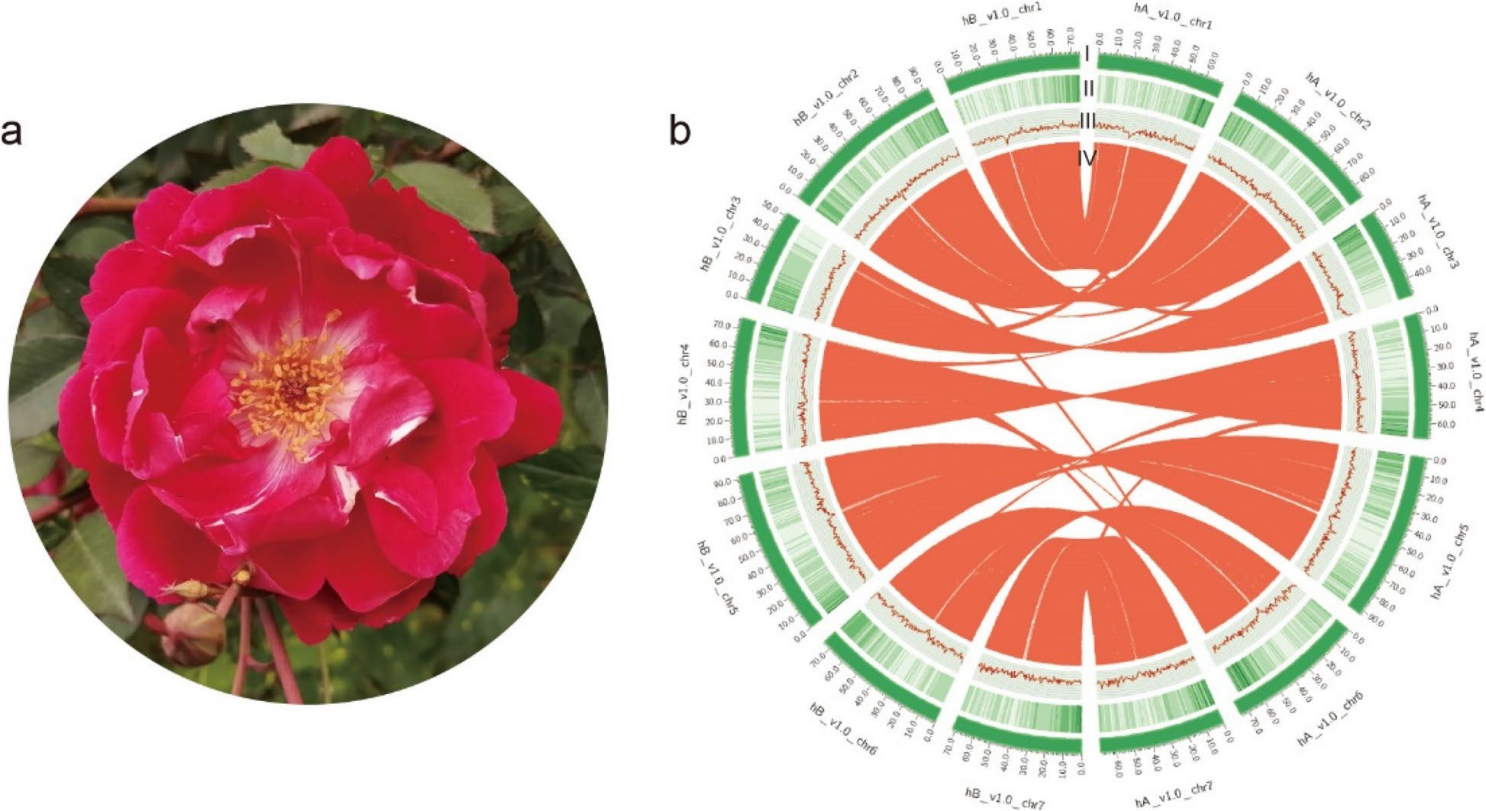
The features of *Rosa chinensis* ‘CH’ genome. **a** The plant of *R. chinensis* ‘CH’. **b** Distribution of ‘CH’ genomic features. (I) Circular representation of the 14 chromosomes of two haplotype genomes, length in Mb, (II) gene density, (III) GC content in 500 kb windows, and (IV) each linking line in the center of the circle connects a pair of homologous genes between the two haplotype genomes of ‘CH’

We generated ~ 25 Gb of PacBio HiFi reads and ~ 34 Gb raw MGI short reads from *R. chinensis* ‘CH’ (2*n* = 14) (Fig. 1a and Table S1). The genome size of ‘CH’ was estimated to be ~ 538 Mb, with a heterozygosity of 2.57% by survey (Fig. S1 and Table S2). We obtained the initial assembly containing two divergent haplotypes with Hifiasm software after removing organelle sequences, resulting in genomes of 518 Mb (hA) and 541 Mb (hB), with contig N50 values of 10.89 Mb and 18.05 Mb, respectively. In addition, we observed that the heterozygous regions were mostly collapsed into single-copy homozygous content of the two genomes by KAT software suggested the correctness of genome phasing (Fig. S2). Most (97.9% and 98.4%) of the genome contigs were respectively anchored to 7 pseudochromosomes by Hi-C data (Table S3), which yielded well BUSCO evaluation (Table 1 and Table S4). To confirm the accuracy of phasing, we used the following strategy: 14 chromosomes were placed in a Hi-C heatmap with strong interaction patterns (Fig. S3); Subsequent remapping efforts demonstrated that high mapping rates for HiFi reads (99.85% and 99.84%, respectively) and MGI reads (98.58% and 98.39%, respectively) (Table S6); GC content and second generational data coverage depth shown the genomes without external pollution (Fig. S4); The quality value were 47.084 (hapA) and 47.415 (hapB); LAIs were 19.93 (hA) and 21.24 (hB); In addition, good collinearity was observed between the two haplotype genomes and between each haplotype genome separately and the previous ‘OB’ genome (Fig. 1b and Fig. S5); The evaluated switch error rate is 5.30% in our assembled genome. Collectively, these results strongly indicated that we obtained two high-quality haplotyped genomes and hapB as the primary haplotype with higher quality.

**Table 1:** Statistics for *R. chinensis* ‘CH’ Haplotype genomes

	HapA	HapB
Contig N50	10.89 Mb	18.05 Mb
scaffolds N50	69.83 Mb	74.66 Mb
Longest contig	92,402,791 bp	92,644,500 bp
Chromosome number	7	7
Number of contigs	342	264
Number of scaffolds	193	176
Genome-size	518,698,571 bp	541,343,551 bp
Rate of anchoring (%)	97.9	98.4
GC content (%)	38.95	38.84
BUSCO_genome (%)	98.4	98.4
TE (%)	59.6	61.83
Number of predicted genes	31,744	32,340
BUSCO_protein (%)	98.6	99.0

We predicted 31,744 genes in hA and 32,340 in hB, and the BUSCO evaluation results showed completeness of 98.6% and 99.0%, respectively (Table 1 and Table S6, 7, 8), proving the high quality of the gene structure prediction results, which were sufficient for further analyses. We found that the predicted genes were mainly distributed at the ends of the chromosomes (Fig. 1b). Among the TEs, 59.60% (hA) and 61.83% (hB) were predicted to be transposable elements, among which Copia LTR-RTs accounted for 14.27% and 13.46% and Gypsy accounted for 12.79% and 12.94%, respectively (Table S9). In addition, 710 (hA) and 677 (hB) transfer RNA, 1,746 and 2,083 ribosomal RNA (rRNA), 667 and 687 small nuclear RNA, and 162 and 153 microRNA (miRNA) genes were predicted using Infernal.

### Genetic variation of the two haplotype genomes in highly heterozygous ‘CH’

**Fig. 2:**
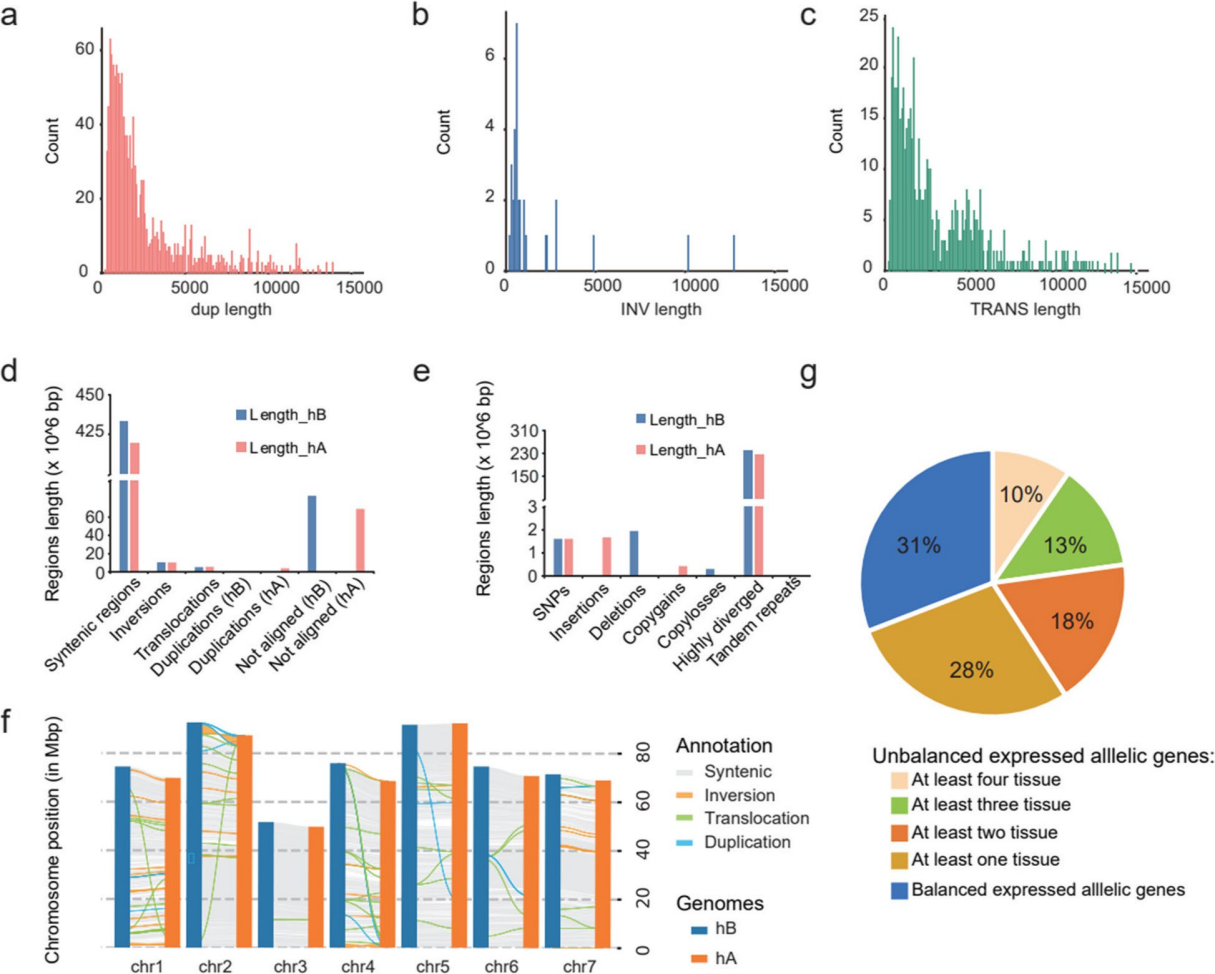
Genetic variation of the haplotype genome in high heterozygous *R. chinensis* ‘CH’. **a**-**c** The length distribution of duplications (**a**), inversions (**b**) and translocations (**c**) between the two haplotypes genomes. **d**-**e** The genomic structural variation characteristics between the two haplotype genomes. hA and hB mean the haplotype A and haplotype B of ‘CH’ genome, separately. f. Collinearity and structural variation distribution between two haplotypes of ‘CH’. g. Differential expression ratios of alleles in four tissues of roots, stems, leaves, and flowers

To evaluate the divergence between the two haplotypes of ‘CH’, we identified genetic polymorphisms between the 7 homologous chromosome pairs. The Synteny and Rearrangement Identifier (SyRI) tool revealed 201 syntenic blocks, 1,605,616 single nucleotide polymorphisms (SNPs), 209,575 indels (1–50 bp insertion and deletion), 59 inversions, 1,169 translocations, 5 tandem repeats, 1,425 duplications and 2,489 large insertion and large deletion (> 50 bp) between the two haplotypes (Table S10–11 and Fig. S6a). What's more, the biggest inversion of chromosome 2 was supported by HiFi reads mapping (Fig. S6b). Among these variations, the lengths of inversions, translocations and duplications were mainly distributed in the around 1,000 bp (Fig. 2a–c). In addition, we identified ~ 83 Mb of unique regions in hB, ~ 69 Mb of unique regions in hA and highly diverse regions in the two ‘CH’ haplotypes (Fig. 2d–f, Table S11). Based on synteny and sequence similarity, 16,889 pairs (33,778 genes, 52.70% of all annotated genes) which contain at least one SNP variation were considered allelic genes, alongside 6,207 “single allele” with identical coding sequences between the two haplotypes. To understand the expression landscape of allelic genes, we analyzed the transcriptome data of four tissues (roots, stems, leaves, and flowers) using the Kallisto pipeline (Fig. S7). A total of 13,971 of allelic genes were differentially expressed in different tissues. Among these expressed allelic genes, 1,342 (~ 10%) pairs unequal expression (*P* < 0.01) between two alleles in all tissues exhibited, as did 3,949 (~ 28%) pairs in at least one tissue (Fig. 2g and Fig. S8). These differentially expression of alleles may be related to the structural variations.

### Phylogenetic evolution

**Fig. 3:**
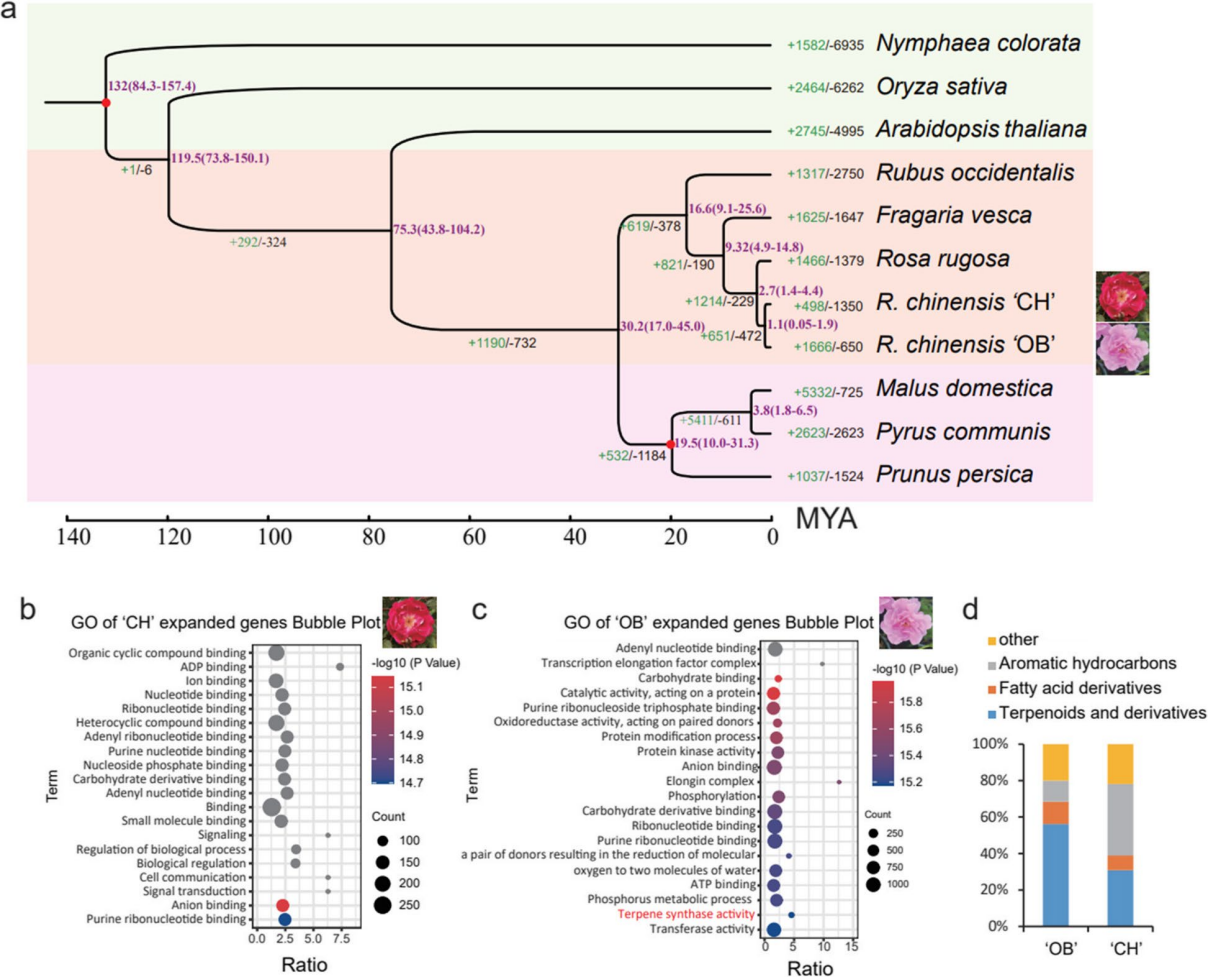
Evolution of the *R. chinensis* ‘CH’ and influence of family gene expansion on the main components of floral aroma. **a** Phylogenetic tree for ‘CH’ hB and ten other eudicot species contained three outgroups. Gene family expansions are indicated in green, and contractions in black; The estimated divergence time (million years ago, MYA) is indicated at each node; numbers in brackets are the 95% confidence intervals (each center is defined as mean value). The red dot represents a calibration point. **b**-**c** Gene Ontology (GO) enrichment analysis of expanded gene families in ‘CH’ (**b**) and ‘OB’ (**c**). **d** Accumulation histogram of four groups of volatile organic compounds of *R. chinensis* ‘OB’ and ‘CH’

To understand the phylogenetic evolution of ‘CH’, we searched for conserved orthologous gene families in the genomes of *Nymphaea colorata*, *Oryza sativa*, *Arabidopsis thaliana*, *Fragaria vesca*, *Malus domestica*, *Prunus communis*, *P. persica*, *Rubus occidentalis*, *R. chinensis* ‘OB’, and *R. rugosa*. Among these species, *N. colorata*, *O. sativa* and *A. thaliana* were selected as outgroups. A total of 681 single-copy orthologous groups were identified with OrthoFinder to construct a species tree of ‘CH’ (hapB/hB) and representative Rosaceae species. ‘CH’ hB and ‘OB’ constituted sister groups belonging to *R. chinensis,* and *R. rugosa* was the closest member of the species. The divergence time of ‘CH’ (hB) and ‘OB’ was estimated to be ~ 1.1 MYA. In addition, that of *R. chinensis* and *R. rugosa* was estimated to be ~ 2.7 MYA (Fig. 3a). After replace genome hB with hA and using the same pipeline, the similar phylogenetic relationship was obtained (Fig. S9). What's more, we identified (498 and 320 respectively) expanded gene families and (1,350 and 604) contracted gene families in ‘CH’ hB and hA (Fig. 3b). Compared to either ‘CH’ hA or hB, there were more expanded gene families in ‘OB’, especially terpene-related gene families (Fig. 3b–c and Fig. S10). In order to confirm whether there was difference of the volatile organic compounds present between the two cultivars, the GC–MS experiment was performed. We found that there were more terpenoids and derivatives in ‘OB’ than in ‘CH’ (Fig. 3d). By analyzing the transcriptome data of ‘OB’ at different flower developmental stages, we found that eight related genes were expressed (FPKM > 1) with increasing pattern of expression during the flower development (Fig. S11). Overall, the eight expanded family genes above in ‘OB’ may contribute to more terpenoids and derivatives in ‘OB’ than in ‘CH’.

### Identification of pigments and differentially expressed genes in red and pink petals

**Fig. 4:**
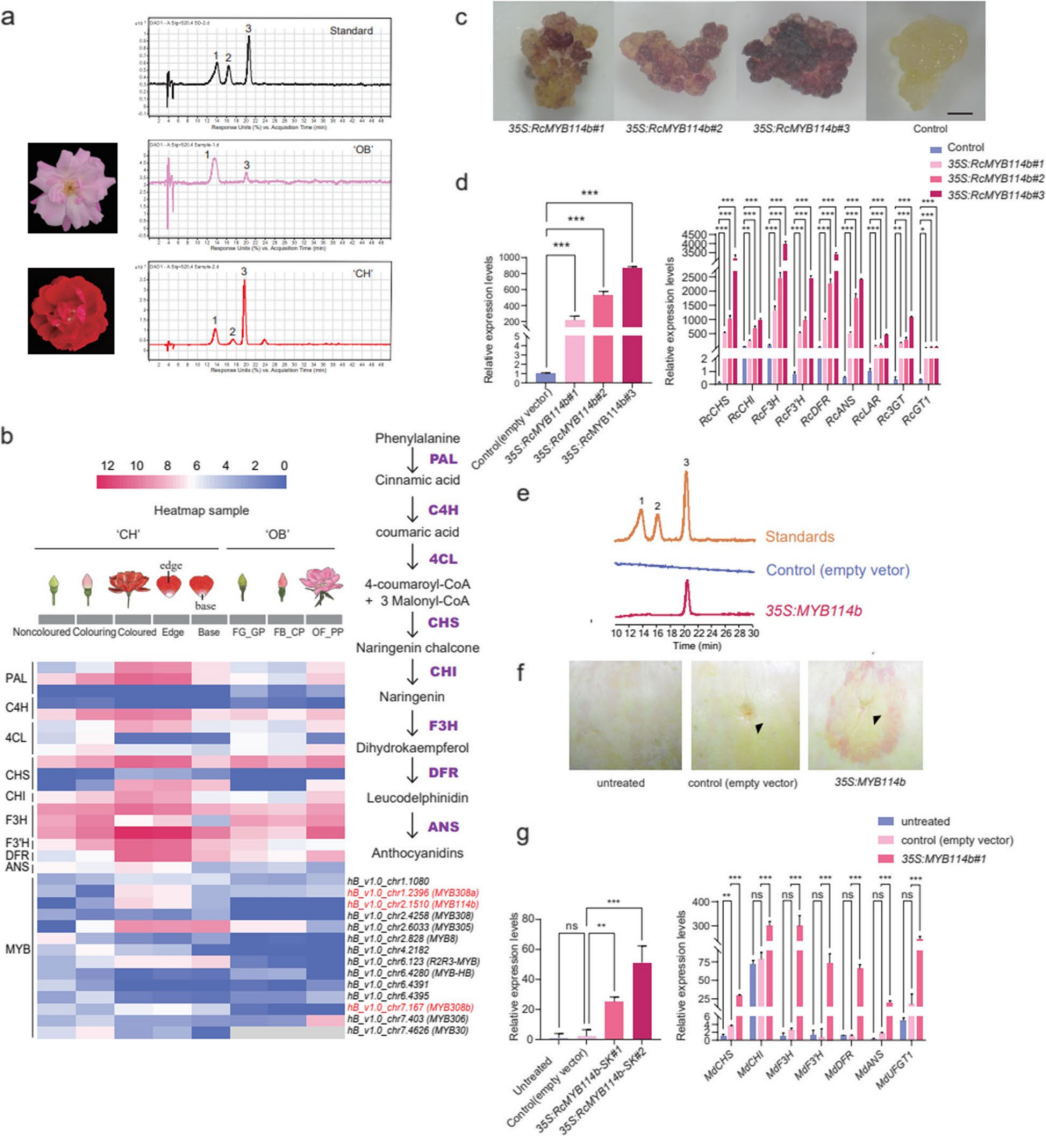
Potential anthocyanin regulatory pathway of red or pink petals in *R. chinensis*. **a** The metabolites were identified by LC_MS in ‘CH’ and ‘OB’ petals. Peak 1–3 represent cyanidin-3,5-diglucoside, pelargonidin-3,5-O-diglucoside and cyanidin-3-glucoside, respectively. **b** Proposed biosynthetic pathway of anthocyanin synthesis in rose. **c** The different *35S:RcMYB114b* transgenic lines of rose turned red. **d** Expression of *RcMYB114b* (left) and anthocyanin biosynthesis pathway genes (right) in different *35S:RcMYB114b* transgenic lines of rose callus. The bars indicate the SD. Stars mean significant differences (*** represents *P* < 0.001, ** represents *P* < 0.01, * represents *P* < 0.05) analyzed by one–way ANOVA. e. LC–MS analysis of *35S:RcMYB114b* transgenic rose callus and control. Peak 1–3 represent cyanidin-3,5-diglucoside, pelargonidin-3,5-O-diglucoside and cyanidin-3-glucoside, respectively. f. The transient expression of *RcMYB114b* in apple fruit. The black triangle represents the site of the injection. g. Expression of anthocyanin biosynthesis pathway genes in *RcMYB114b* transgenic lines of apple. The bars indicate the SD. Stars mean significant differences (*** represents *P* < 0.001, ** represents *P* < 0.01, * represents *P* < 0.05) analyzed by one–way ANOVA

To explore the molecular mechanism underlying the colour difference between red and pink petals, the metabolites of these petals were identified. Cyanidin-3,5-diglucoside and cyanidin-3-glucoside were found to be the main anthocyanin components of the red and pink petals (Fig. 4a). Cyanidin-3,5-diglucoside was the predominant component in the pink petals of ‘OB’, while cyanidin-3-glucoside was the predominant component in those of ‘CH’ (Fig. 4a).

By analyzing the transcriptome data of petals (noncoloured petals vs. colouring petals, colouring petals vs. coloured petals, and red petal regions vs. the white heart petal regions) in ‘CH’ and ‘OB’, we found that the expression of anthocyanin biosynthesis pathway genes, including *phenylalanine ammonia-lyase* (*PAL*), *chalcone synthase* (*CHS*) and flavanone 3-hydroxylase (*F3H*), was higher in red petals than in pink petals and in coloured petal regions than in the white heart region of petals (Fig. 4b). In addition, there were some related differentially expressed transcription factors (TFs) identified between different petal colouring stages, such as *MYB*, *bHLH*, ethylene response factor (*ERF*) (Fig. S12). Among these genes, the expression of *RcMYB114b*, encoding MYB transcription factors (Fig. 4b and Fig. S13), showed an upwards trend during the process of petal colouring development in ‘CH’, while, *RcMYB308a* and *RcMYB308b* mainly in ‘CH’ coloured stage. And their expression trend was confirmed by real-time quantitative PCR (qRT‒PCR) in ‘CH’ and ‘OB’ (Fig. S14). Notably, based on the variation between identified alleles between two haplotypes and two materials (Table S10-S11 and Fig. S15), we found that there were many variants between alleles of *RcMYB114b* promoter, suggesting that *RcMYB114b* may contribute more to the formation of petal colour.

### *RcMYB114b* contributes to the formation of petal colour

To investigate the function of *RcMYB114b*, the coding sequences (CDSs) of *RcMYB114b* amplified from ‘OB’ and ‘CH’ were compared, and their amino acid sequences were identical. Overexpression of *RcMYB114b* in ‘OB’ calli resulted in a colour change, with the transgenic calli turning red due to cyanidin-3-glucoside accumulation, while the control calli did not change colour (Fig. 4c–e). Transcriptome sequencing of the transgenic lines of rose calli revealed 2,832 differentially expressed genes (DEGs) compared to the control calli and these DEGs were associated with various KEGG enrichment terms (Fig. S16). Among the DEGs, key genes involved in the flavonoid biosynthesis pathway, such as *CHS*, *F3H*, *flavonoid 3'-hydroxylase* (*F3’H*), *DFR*, and *leucoanthocyanidin reductase* (*LAR*), and transcription factors *RcMYB114b* and *RcbHLH95*, showed significant changes in expression level (Fig. S16c, d). Moreover, the qRT‒PCR results validated the upregulation of gene expression for *RcCHS*, *chalcone isomerase* (*RcCHI*), *RcF3H*, *RcF3’H*, *RcDFR*, *anthocyanidin synthase* (*RcANS*), *RcLAR* and *anthocyanidin 3-O-glucosyltransferase* (*Rc3GT*) in transgenic calli, consistent with the RNA-seq results (Fig. 4d). Furthermore, the transient transformation of *RcMYB114b* into *M. pumila* (Rosaceae) demonstrated similar changes: the injection site turned red, and related gene (*MdCHS*, *MdCHI*, *MdF3’H*, *MdDFR*, *MdANS* and *lavonoid 3-O-glucosyltransferase* (*MdUFGT1*)) expression was upregulated, while the empty vector injection site showed no change in colour (Fig. 4f–g). Collectively, these results indicate that *RcMYB114b* functions as an activator of anthocyanin biosynthesis, influencing cyanidin-3-glucoside accumulation in petals by activating the expression of genes involved in anthocyanin synthesis pathway and regulating the formation of red colouration in petals.

To clarify why *RcMYB114b* expression was different between ‘OB’ and ‘CH’, the promoter sequences of *RcMYB114b* in ‘OB’ and ‘CH’ were identified based on the haplotype genomes and amplified, and we found that there was only one promoter genotype of *RcMYB114b* in ‘CH’, named *RcMYB114bpro*^*long*^, while in ‘OB’ there were two genotypes. One was identical to that in ‘CH’ and the other had multiple SNPs, 6 indels and 2 large deletions, and was named *RcMYB114bpro*^*short*^ (Fig. S17). Transient promoter activity assay was performed and the results showed that *RcMYB114bpro*^*long*^ was more active than *RcMYB114bpro*^*short*^ (Fig. S18), which was the reason for the different expression levels of *RcMYB114b* in ‘CH’ and ‘OB’.

### Multi-TFs affect the response of rose petal number and size to high temperature

**Fig. 5:**
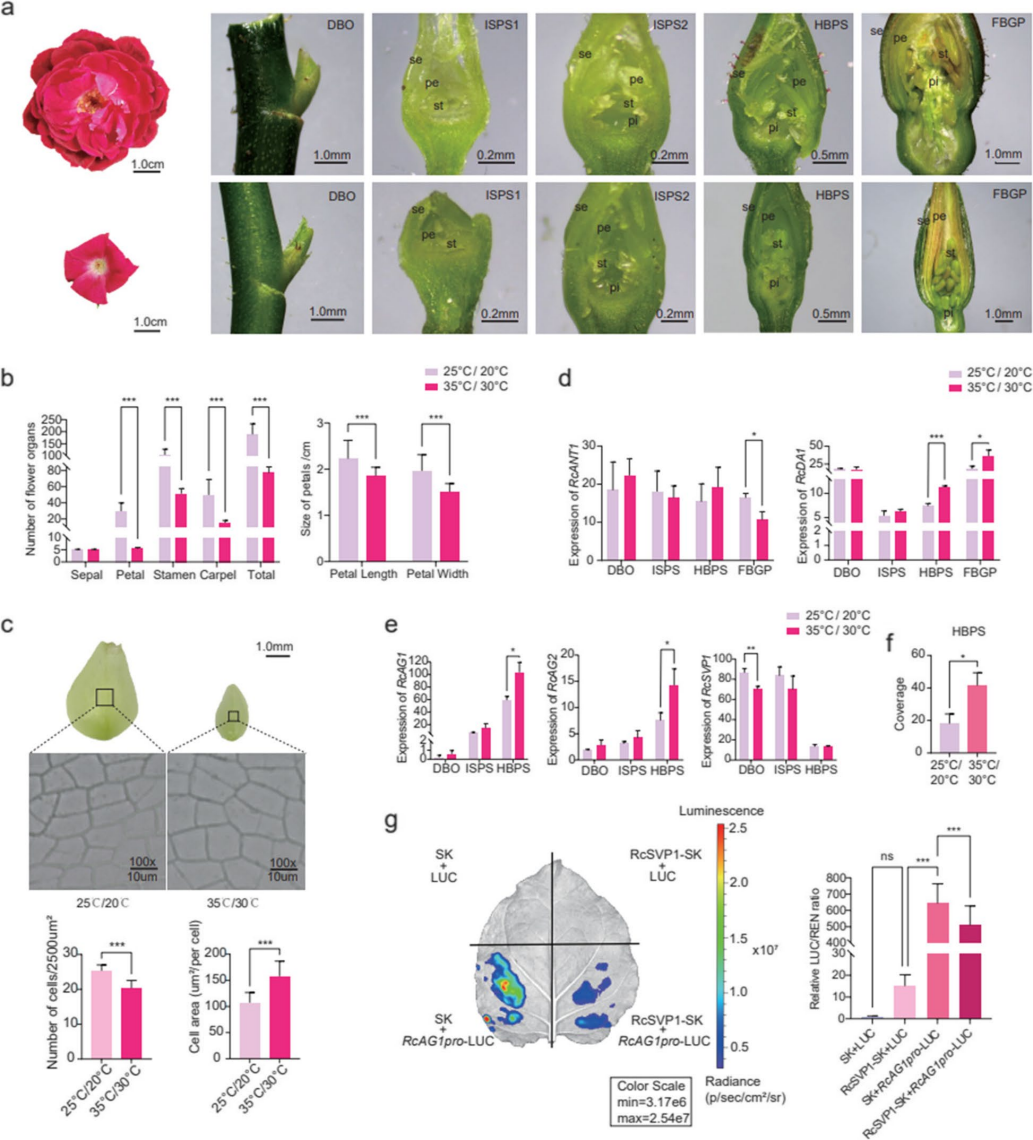
Changes in the number and size of petals in *R. chinensis* ‘CH’ under high temperature treatment. **a** Flower morphology at different stages under high temperature (downer) and normal temperature (upper) treatment. **b** Statistics of the number and size of floral organs under different temperature treatments. Stars (***) mean significant differences (*P* < 0.001) analyzed by two–way ANOVA. **c** The cell density middle of petals under high temperature (right) and normal temperature (left) treatment. **d**-**e** The expression of *RcANT1*, *RcDA1*, *RcAG1*, *RcAG2* and *RcSVP1*. Stars mean significant differences (*** represents *P* < 0.001, ** represents *P* < 0.01, * represents *P* < 0.05) analyzed by T-test. f. The coverage of *RcAP2L*^*wt*^ exon 10th with miR172 binding site under high temperature treatment. Stars mean significant differences (* represents *P* < 0.05) analyzed by T-test. g. The dual‐LUC assays. Red represents higher signal intensity and blue represents lower signal intensity. The bars indicate the SD. Stars (***) mean significant differences (*P* < 0.001) analyzed by one–way ANOVA

During the cultivation process, we found that the petal number of ‘CH’ plants was obviously affected by environmental factors. When ‘CH’ plants were treated at high (35 / 30 ℃) and normal temperatures (25 / 20 ℃), it was very interesting to see that the double-flower phenotype (more than 25 petals) of ‘CH’ plants observed at normal temperatures shifted to a single-flower phenotype (~ 5 petals), with smaller petals, at high temperatures (Fig. 5a–c). In ‘OB’, however, there was no significant reduction in petals (Fig. S19). While, stamen and carpel number were affected both in ‘CH’ and ‘OB’ under high temperature treatment.

In addition, we observed a significant decrease in the number of abaxial epidermis of petals under high temperature treatment (25.33 ± 1.67 to 20.33 ± 2.23), accompanied by a significant increase in petal cell area (107.23 ± 19.44 µm^2^ to 157.82 ± 28.99 µm^2^) at FBGP, potentially contributing to the reduction in petal size (Fig. 5c). Then, we collected samples from the four stages for RNA sequencing. Through weighted correlation network analysis (WGCNA) (Fig. S20, 21), we identified four modules (Blue, Darkgrey, Lavenderblush3 and Darkseagreen) potentially associated with petal and stamen primordium development, which were further analyzed using Gene Ontology (GO) analysis (Fig. S22). Among the genes of the modules, the expression of *RcANT1* (homologous to *AINTEGUMENTA* in *Arabidopsis*), reported to regulate cell proliferation (Krizek 1999, Delgado-Benarroch et al. 2009) was downregulated after high-temperature treatment in FBGP, while the expression of the *RcDA1* gene (homologous to *DA* in *Arabidopsis*, which restricted the duration of the cell proliferation phase of floral organ growth (Li et al. 2008) was upregulated (Fig. 5d). The changes in *RcANT1* and *RcDA1* expression may contribute to the cell proliferation of petals in response to high temperature in rose.

Furthermore, we conducted a comparative analysis of DEGs between the two key cell proliferation groups (25 ºC ISPS_vs_HBPS and 35 ºC ISPS_vs_HBPS). There were 842 common DEGs in the two groups containing 13 MADS box genes related to flower development and 1 class A gene *RcAP2L* (Fig. S23 and Table S12). Among them, the expression of *RcAG1* increased with the formation of flower organ primordia and was higher in ISPS and HBPS under high-temperature treatment than under normal temperatures. *RcAG2* also had a similar expression pattern, but its expression level was lower than *RcAG1* (Fig. 5e). The expression of *RcSVP1* gene was significantly down-regulated. Dual-luciferase assays indicated that *RcSVP1* involved in inhibiting the expression of *RcAG1* and affect petal numbers under high temperature (Fig. 5g). However, it indicated that *RcSVP1* could not directly bind to the promoter of *RcAG1* (Fig. S24). The two *RcAP2L* alleles of ‘CH’ were obtained, one contained about 10 kb TE insertion, while the other did not. Both have miR172 binding sites in gene (Fig. S25). Based on transcriptome data, five splicing variants of *RcAP2L* in CH were obtained (Fig. S26). Notably, the level of reads coverage of miR172 binding site on exon 10th under high temperature environment was significantly higher than that under normal temperature at HBPS stage (Fig. 5f), which may result in stronger inhibition *RcAP2L* translation in high-temperature environments by miR172.

## Discussion

### Haplotype genomic information provided a new insight to trait diversity of R. chinensis

To maintain consistent diversity/quality of ornamental traits, rose is usually propagated vegetatively to maintain a state of high genomic heterozygosity. At present, there are some excellent germplasm resources for rose cultivars. However, if we seek to further improve these germplasms through hybridization, the original excellent traits may be lost, which severely limits the improvement of rose. Previous studies have found that molecular breeding using genomic information can improve breeding efficiency (Li and Zhang 2013, Liu et al. 2022). Especially, the haplotype genome is obtained to fully preserve the integrity of genetic information and greatly accelerate the breeding process such as in potato (Zhou et al. 2020b) and *Bletilla striata* (Jiang et al. 2022). In this study, two haplotype-resolved genomes of *R. chinensis* ‘CH’ were first assembled. The genetic variation and differential expression of alleles between the two haplotypes were evaluated. All of these results provide guidance for exploring the regulatory mechanisms of important traits and assessing haplotype diversity to accelerate the improvement of rose in the future. To date, the accuracy phasing and accuracy assembly of highly heterozygous species have still posed significant challenges with current sequencing technologies. In the future, a ‘CH’ genetic map will be useful to validate the phasing. With advancements in sequencing technology, and the update of assembly software such as hifiasm, these advancements pave the way possibilities in assembling the genomes of highly heterozygous flowers or polyploid species, enabling the acquisition of more comprehensive and precise typing genome information.

Allelic variation confers the imbalanced expression of alleles, and contribute to trait diversity. For example, in apple, the presence of the TE insertion in the allele of *MYB* was positively associated with ASE and petal color (Tian et al. 2022). In Lettuce, the alleles of *Red Lettuce Leaves 1* (*RLL1*) gene, a *bHLH* transcription factor, with 5 bp base deletion or not, regulated the leaf colour red or green (Su et al. 2020). In our study, we propose that *RcMYB114b* is an anthocyanin activator that is similar to those found in many plants, such as citrus, sweet cherry, and snapdragon (Schwinn et al. 2006, Jin et al. 2016, Huang et al. 2018). But it's interesting that in rose *RcMYB114b* was found to control the petal colour change from pink to red due to variations in promoter sequences. It was further proven that the allelic sequences may have different functions, so more comprehensive sets of clean haplotype genome information are needed to elucidate the molecular mechanism of important ornamental traits.

### Complex and diverse mechanisms for regulating flower color and pattern of roses

Anthocyanins are important metabolites that regulate the formation of petal color. Previous studies have reported that S6 subgroup of MYB involved in anthocyanin accumulation, contained 5 genes (*RcMYB113a*, *RcMYB113b*, *RcMYB113c*, *RcMYB114a* and *RcMYB114b*) (Li et al., 2021). Three of them (*MYB113a, RcMYB11*3c called *RcMYB114* in previous reports, and *RcMYB114a*), have been reported (Lin-Wang et al., 2010; Li et al. 2022, Yan et al. 2023). In our study, another new gene *RcMYB114b* was identified*.* These reported showed that although these five genes belong to the same subfamily, they not only regulate different metabolite accumulation, but also have different expression sites/periods. For example, *RcMYB114a* (Yan et al. 2023) and *RcMYB114b* promoted cyanidin-3-glucoside accumulation. While *RcMYB113c* promoted cyanidin-3-O-sophoroside accumulation (Li et al. 2022). In contrast, another homolog of *RcMYB113a*, *FaMYB10*, has been reported in strawberry, and overexpression of *FaMYB10* promoted the accumulation of cyanidin-glucoside, pelargonidin-glucoside and pelargonidin (Lin-Wang et al., 2010). In addition, *RcMYB114a* effected the accumulation of anthocyanin in response to light/dark, while *RcMYB114b* cannot. Interestingly, their expression pattern also was significantly different. The expression of *RcMYB114a* was up-regulated at the flower in early opening stage and highest at the fully open flower stage, whereas the expression of *RcMYB114b* was highest at the flower in early opening stage and down-regulated at the fully open flower stage. Different from above, *RcMYB11*3c was largely unexpressed (Yan et al. 2023). In addition, different expression patterns of homologous genes were also found in strawberry, which may affect fruit color (Lin-Wang et al., 2010). All these founds indicate that the S6 subgroup genes may have been newly functionalized to regulate the accumulation of different substances, and subfunctionalized to regulate the same substances accumulation, but at different sites and periods. The discovery of a new gene *RcMYB114b* in our study provides new insights into the functional differentiation of the S6 subgroup genes in rose.

High temperatures have an important impact on flower yields and morphology of rose. In our study, under high-temperature treatment, we found that not only the number of petals but also petal size changed in the proliferation stage of petal cells. Previous studies not only showed *DA1* (Li et al. 2008, Gao et al. 2021), *ANT1* (Krizek 1999, Randall et al. 2015), and Class -A (*AP2* and *AP1*), -B (*AP3* and *PISTILLATA*), -C (AG) genes in volved in petal development, but also showed gene epigenetic regulation play important roles in petal number changes (Law and Jacobsen 2010, Jones 2012). For example, reduced expression of *AG* promoted the formation of double petal flowers, and increased methylation of the *RhAG* promoter at low temperature (15 / 5 ℃) has been linked to a higher number of petals in roses (Ma et al. 2015). In addition, the transcription may also regulate the petal number (François et al. 2018). Consider the miR172 binding site was the key factor effecting petal number rather than one isoform’s expression. In our results, we detected the level of reads coverage of miR172 binding site on exon 10th was higher under high temperature environment. As previous study found that the more expression of miR172 binding site, it could more decrease the RcAP2L protein to effect petal number (Chen 2004). And interestingly, under high temperature treatment, ‘CH’ and ‘OB’ showed different phenotypes which containing different genotypes. In a nutshell, all these indicated that flower development under high-temperature treatment involves a very complex regulatory network, and more studies are required to clarify this in the future.

## Materials and methods

### Plant materials

*R. chinensis* ‘CH’ and ‘OB’ are widely recognized old rose cultivars. Varieties selected from these two cultivars for this study were growing in the same experimental field under natural conditions at the Flower Garden of Huazhong agricultural University. The leaves of ‘CH’ were subjected to DNA extraction for genome sequencing, and extracts of its young stems, flower buds, leaves and young roots were used for genome annotation.

To explore the formation of petal colour, samples of petals in three stages (noncoloured petals, colouring petals, coloured petals) and two petal parts (the red region and the white heart region of coloured petals) were collected for RNA-seq. A total of five samples were collected from ‘CH’ for analysis of the anthocyanin regulatory pathway. The different petal stages selected were consistent with the published petal sampling stages of ‘OB’ (Han et al. 2017). The petals of six flowers at each developmental stage were collected as one biological replicate, with a total of three biological replicates for RNA-seq, anthocyanin extraction and liquid chromatography-mass spectrometry (LC–MS) analysis.

To analyze the effect of high-temperature treatment on rose flower development, sixty individual ‘CH’ plants were pruned and then randomly placed into two incubators with two different parameter settings: (1) 16 / 8 h day/night at 35 °C / 30 °C and (2) 16 / 8 h day/night at 25 °C / 20 °C. After 50 d, the four different stages of flower buds: the vegetative meristem stage (DBO); the initiation stages of petals/petal-like structures and stamens/stamen-like structures (ISPS); the stage in which the hypanthium starts to sink below the perianth and stamens (HBPS); and the stage of flower buds with young noncoloured petals (FBGP), were observed individually and were collected under a microscope (SOPTOP SZN, Yuyao, China) in three biological replicates for RNA-seq.

The numbers of sepals, petals, stamens and carpels were counted in at least thirty flowers under heat treatment and control conditions. Statistical analysis was performed using two–way ANOVA along with the least significant difference test using GraphPad Prism version 9.0, and *P* < 0.05 was considered significant. Transgenic callus of the same line was snap frozen in liquid nitrogen and stored at − 80 °C in the refrigerator until transcriptome sequencing, quantitative detection, pigment extraction and LC–MS.

### Microscopic examination and cell counting

Cell photography and counting were performed as described by previous reports (Dewitte et al. 2007) with some modifications. In short, petal samples at stage of flower buds with young noncoloured petals (FBGP) in the above two incubators were taken as slices at 50% of the petal length from the petal top. The slices were embedded in 3% agarose gel (Biosharp, Beijing, China) and 60 μm sections were cut on a semi-automatic Leica VT1200 vibrating blade microtome (Weztlar, Germany). The abaxial epidermis cells of the slices were photographed using a Novel digital microscope (NLCD500, Nanjing, China). Fifteen flowers were used in each treatment. Numbers of cells were counted in a visual field of 50 × 50 μm^2^. The area of cell was measured in ImageJ software. Statistical analysis was performed using T-test by GraphPad Prism version 9.0, and *P* < 0.05 was considered significant.

### Genome sequencing, assembly and anchoring

MGI data sequencing. The healthy young leaves of *R. chinensis* ‘CH’ were quickly frozen in liquid nitrogen and stored in a − 80 °C freezer for MGI and HiFi sequencing. A library with an insert size of 150 bp was produced using MGI to survey the genome.

HiFi data sequencing. Genomic DNA was prepared by purification with a QIAGEN® Genomic kit (QIAGEN) according to the manufacturer’s instructions. More than 10 μg of genomic DNA was used to construct ~ 20-kb-target-size Sequel SMRTbell libraries in circular consensus sequencing (CCS) mode for PacBio HiFi sequencing.

Hi-C data sequencing. A Hi-C library was constructed from young leaves of ‘CH’, and sequenced via the Illumina NovaSeq platform. Low-quality sequences and adaptor sequences were filtered out, and the unique mapped paired-end reads were extracted by mapping the clean paired-end reads to the draft assembled sequence.

To estimate the size of the genome, the 21-mer frequency distribution was analyzed using Jellyfish (v2.3.0) (https://github.com/jamesturk/jellyfish). Hifiasm (v0.16.1-r375) software (Cheng et al. 2021) was used to assemble the genome of two haplotypes of *R. chinensis* ‘CH’ using HiFi reads by diploid phased assembly using the parameter “hifiasm -o asm –h1 HIC_1.fq.gz –h2 HIC_2.fq.gz genome.fastq.gz”. The resulting contigs were aligned to rose plastid genomes using BLASTn (v2.9.0), and the contigs in which more than 95% identity with plastid genome were filtered. Then, the clean data were used for two rounds of error correction by Pilon (v1.23). Hi-C reads were mapped to the assembled scaffolds using Juicer (v1.6) (Durand et al. 2016) with the default parameters, and 3D-DNA (Dudchenko et al. 2017) was used to cluster and order them into 14 chromosomes with parameters “-r 0”. After manual adjustment by Juicebox v1.11.08, we obtained the two haplotype genomes (hA and hB), which were fully resolved at the chromosomal level. We used the following strategies to assess the quality of the genomes: (1) The BUSCO (v4.0.6) database (eudicotyledons_odb10 database) was used to assess the continuity of the assembly with parameters “-m geno”. (2) The heterozygous regions were detected by KAT software and the MGI and HiFi reads were mapped back to the genome. (3) The mapping rate as well as genome coverage of sequencing reads were assessed using samtools v1.7. (4) The Quality Value score was uesd to evaluate this genome by Merqury (Rhie et al. 2020). (5) Whole-genome with fourteen chromosomes Hi-C contact heatmaps were plotted using ALLHIC. (6) The chromosomes of ‘CH’ were aligned to the OB reference genome (Raymond et al. 2018) using MUMmer v3.1 with default parameter (‘OB’ genome as reference genome and ‘CH’ genome as query genome). (7) The switch errors were evaluated by the calc_switchErr (https://github.com/tangerzhang/calc_switchErr).

### Annotation of repetitive sequences and protein-coding genes

TEs in ‘CH’ were identified with the an extensive de novo TE annotator pipeline (Ou et al. 2019) with parameter “–anno 1 –force 1 –debug 1 –sensitive 1”, which combines the widely used RepeatModeler package with Repbase and seven other structure-based methods for de novo LTR identification.

Protein-coding genes for each haplotype genome were predicted using EVidenceModeller (Haas et al. 2008) by integrating three strategies: ab initio gene predictions; RNA-seq read mapping; and protein homology. Ab initio gene predictions were generated by AUGUSTUS v3.3.3 (Stanke et al. 2006), SNAP v2013–02–16 (Korf 2004), and GlimmerHMM v3.0.4 (Majoros et al. 2004). For homology-based prediction, the protein sequences from eight species, including *Arabidopsis thaliana*, *Oryza sativa*, *Vitis vinifera* and the Rosaceae members *R. chinensis* ‘OB’, *R. chinensis* ‘OB’ _DH, *P. persica*. v2, *Pyrus betulifolia*, and *F. vesca* v4 (https://www.rosaceae.org/tools/jbrowse)*,* were integrated by Exonerate v2.2.0 (http://www.ebi.ac.uk/~guy/exonerate/) and AUGUSTUS. For transcriptome-based prediction, RNA-seq data were mapped using HiSAT2 v2.1.0 (Kim et al. 2015) and then assembled with PASA v 2.4.1 (Haas et al. 2003). The final annotation of protein completeness was evaluated with BUSCO.

All predicted proteins were aligned against the UniProt SwissProt and NR databases. The functions of the best-matched proteins were assigned to the predicted proteins. Functional annotation was performed using InterProScan v5.21 (Jones et al. 2014). GO terms were assigned according to the InterPro classification.

### The overall view of the comparisons and diversity analysis between the two haplotypes

To identify the variation between the two genomes, we used SyRI coordinates to align them pairwise and identify variations such as candidate inversions, intra- and inter-chromosomal translocations (Goel et al. 2019). To identify the homologous regions between two haplotypes, the syntenic blocks were constructed based on well-aligned genes (CDSs) using MCScanX package. We screened for allelic genes based on the following criteria: (1) paired regions must be located on homologous chromosomes within syntenic blocks; (2) The gene has another best homologous gene on another haplotype and each of the gene pairs should be a reciprocal blast best hit; (3) The coding sequence alignment needed to exhibit at least one SNP. Genes that satisfied these specific criteria were classified as allelic genes. If genes had identical coding sequences within syntenic blocks between the two haplotypes, they were labeled as “single allele”.

### Allelic genes expression analysis in different tissues

We performed allelic gene expression analysis using the allele-specific mapping of Kallisto which was used in the comparison of homologous expression in polyploid wheat (Ramírez-González et al. 2018) and potato (Zhou et al. 2020b), and we used the same software to obtain the expression levels, in transcripts per million (TPM), of genes on both haplotypes based on RNA-seq data from different tissues (roots, stems, leaves and flowers). These genes with average TPM (value > 1) for any of tissues were selected as expressed genes. We then tested for expression differences between allelic genes using the T-test with a significance threshold of *P* < 0.01.

### Phylogenetic evolutionary analysis

The species phylogenetic tree of *N. colorata*, *O. sativa*, *A. thaliana*, *F. vesca*, *M. domestica*, *P. communis*, *P. persica*, *R. occidentalis*, *R. chinensis* ‘OB’ and *R. rugosa* was constructed based on single-copy homologous genes with OrthoFinder (Emms and Kelly 2015). The hA and hB genomes of *R. chinensis* ‘CH’ was separately selected for the construction of the species phylogenetic tree. The time calibrations from the TimeTree database (http://www.timetree.org/) were used to estimate divergence times. The single-copy gene sequences were aligned with MAFFT (Katoh and Standley 2013). They were subsequently used to construct a maximum likelihood tree, and the 95% confidence intervals of divergence times were obtained with MCMCtree in the PAML package (Yang 2007).

### Transcriptome analysis

Total RNA was isolated using the EASYspin Plant RNA extraction kit (Aidlab, Beijing, China) following the manufacturer’s protocol. All libraries (three biological replicates for every sample) were sequenced on the BGI platform. Transcriptome data for nine samples of different petal stages of ‘OB’ were downloaded from NCBI (PRJNA351281) (Han et al. 2017). After quality control and the removal of low-quality and adapter-containing reads with Trimmomatic–0.36, the clean data were aligned to the hB genome of ‘CH’ and ‘OB’ using HiSAT2 v2.1.0 (Kim et al. 2015). For accurate quantification of homologous gene expression, only the unique mapping reads were kept for further analysis. Transcript levels were normalized using the fragments per kilobase per million mapped reads (FPKM) method and coverages of exon were calculated with StringTie v1.3.5 (Pertea et al. 2016).

Differential expression analysis of two conditions/groups was performed using the DESeq2 (Love et al. 2014). The resulting *P*-values were adjusted using the Benjamini and Hochberg’s approach for controlling the false discovery rate. Genes with an adjusted *P*-value < 0.05 (log_2_FoldChange > 2 | log_2_FoldChange < -2) found by DESeq2 were assigned as differentially expressed. The Venn diagram and kyoto encyclopedia of genes and genomes (KEGG) enrichment diagram were performed using R software.

### Volatile organic compound collection and analysis

Headspace solid-phase microextraction–gas chromatography‒mass spectrometry (HS-SPME–GC‒MS) technology was used to extract and identify volatile organic compounds. Scent collection was performed in this study according to previous study (Zhao et al. 2016). Freshly picked rose flowers samples of approximately 0.7 g were weighted and then added to a collection bottle. Five independent replicates were performed at the “coloured” stage of *R. chinensis* ‘OB’ and ‘CH’ petals. After water bath and extraction, the fibre was inserted into a GC injection port and then subjected to GC–MS analysis. For chromatographic separation, He (99.999%) was used as the carrier gas. The starting temperature of the column was 40 °C. After a 4 min hold, the temperature was increased to 160 °C at 2 °C/min. Subsequently, the temperature was increased to 250 °C at 15 °C/min with a final holding time of 5 min. The temperature of the transfer line to the mass spectrometer was set at 220 °C. The mass range was 50 to 650 m/z, and full-scanning mode was employed. Xcalibur software (Thermo, Waltham, US) was used for the analysis of mass spectra. M/Z values were matched with those of the National Institute of Standards and Technology (NIST) library. The substances were classified according to the published classification (Zhou et al. 2020a), and the peak area of each type of substance was quantified to compare the proportions of the contents of each substance type.

### Genetic transformation in rose calli

Calli of the ‘OB’ rose cultivar were used for genetic transformation, and the CDS of *RcMYB114b* was amplified and cloned into the pRI 101-AN vector using corresponding primer sequences (Table S13). The constructs of *35S:RcMYB114b* and vector-control were transformed into *Agrobacterium tumefaciens* GV3101 and then transformed into rose calli as previously reported (Vergne et al. 2010) but with minor changes. Briefly, embryos were bulk wounded with sterile sand. The cells were co-cultivated for 1 h at 100 rpm at room temperature. 2 d later, the embryos were washed several times. Approximately 10 d after transformation, embryos were transferred to embryogenic callus maintenance medium supplemented with kanamycin (60 mg·L^−1^) and Timentin (150 mg·l^−1^) and subcultured every 3–4 weeks.

### Transient expression assay in apple fruit

A transient gene expression assay was employed for the overexpression of *RcMYB114b* in apple fruit. The overexpression vector was obtained by cloning the sequence of *RcMYB114b* from the cDNA of ‘CH’ using primer sequences (Table S13), and fused into the pGreenII 62–SK vector. The empty SK and RcMYB114b–SK constructs were then introduced to *A. tumefaciens* strain GV3101 cells. The injections were carried out using a 1 ml needleless syringe as described previously (Li et al. 2012). The injected apples were kept in the dark at 23 °C overnight and were subsequently treated with constant light (100 μmol·m^−2^·s^−1^) for colour development.

### qRT‒PCR analysis

Total RNA from the ‘CH’ and ‘OB’ flower samples, the apple and rose calli of control and transgenic lines was extracted for qRT–PCR. TRUEscript RT MasterMix (Aidlab, Beijing, China) was employed for reverse transcription using 1 μg of RNA. The generated cDNA was a template for qRT–PCR assays with gene-specific rose (Table S13) and apple (Han et al. 2010, An et al. 2018, Kang et al. 2022) primers using Taq Pro Universal SYBR aPCR Master Mix (Vazume, Nanjing, China). The *RcACTIN* (Dubois et al. 2012) and *MdACTIN* (Li et al. 2012) genes were used as internal quantitative controls to normalize samples. The relative expression values were calculated using the comparative CT (2 − ^ΔΔ^CT) method. Statistical analysis was performed via one–way ANOVA along with the least significant difference test using GraphPad Prism version 9.0, and *P* < 0.05 was considered significant.

### Anthocyanin extraction and LC–MS analysis

The anthocyanin metabolites of rose ‘CH’ and ‘OB’ petals and transgenic calli were measured. The samples were powdered in liquid nitrogen. Then, a 0.2 g sample was weighed, placed in an ultrasonicator bath for 30 min with 2 ml of acidic methanol solution (70:27:2:1; v/v, CH_3_OH: H_2_O: HCOOH: CF_3_COOH), and maintained in the dark at 4 °C overnight) (Gao and Mazza 1994, Liu et al. 2016). The extract was centrifuged (12,000 rpm, 10 min, 20 °C), and the supernatant was filtered through 0.22 μm syringe filters (Coolwind, Guangzhou, China) into a 2 ml amber screw autosampler vial (Jiedao, Jiangsu, China). LC–MS analysis was carried out on an Accurate–Mass Q–TOF LC/MS 6520 (Agilent, California, USA). A 150 mm × 4.6 mm, i.d., 4.6 μm, HC–C18(2) column (Agilent, California, USA) was used. Referring to a reported method (Liu et al. 2016), 0.5% aqueous formic acid (A) and acetonitrile (B) were used as mobile phases. The gradients, column temperature, injection volume, and flow rate were programmed as described in a previous report. The ionization of anthocyanin was achieved with an ESI source in positive mode, and the parameters were set followed a previous report (Wan et al. 2018). The anthocyanin chromatograms were extracted at 540 nm.

### Dual-luciferase assays

The promoter fragments of *RcAG1* (1,445 bp upstream of start codon) were inserted into the pGreenII 0800–LUC vector to construct *RcAG1pro:LUC*. The open reading frame (ORF) of *RcSVP1* was inserted into the pGreenII 62–SK vector to construct *35Spro:RcSVP1* (Table S13). The dual-luciferase assay followed the protocol described previously in *Nicotiana benthamiana* (Hellens et al. 2005, Sparkes et al. 2006). Firefly luciferase and Renilla luciferase were assayed 3 d after injection using a Dual-Luciferase Reporter Assay Kit (Promega, Madison, USA). Three independent experiments with a minimum of six replicates were performed to verify the results. Photographs were taken by using an IVIS Spectrum in vivo imaging system (PerkinElmer, Waltham, USA).

Remaining method descriptions are included to Supplementary Notes.

### Supplementary Information

**Additional file 1: Fig. S1.** K-mer analysis for estimating the genome size of R. chinensis ‘CH’ indicating high heterozygosity. **Fig. S2.** The plot depicts the K-mer spectra of the haplotype assembly. **Fig. S3.** Hi-C interaction heatmaps of the two haplotypes (hap A and hap B) in R. chinensis ‘CH’. **Fig. S4.** Sequence depth and GC content for the two haplotypes in R. chinensis ‘CH’. **Fig. S5.** Dot plots with respect to the ‘OB’ genome reference for the two haplotypes assemblies of ‘CH’ respectively.**Fig. S6.** Collinearity of genomes from ‘CH’ and ‘OB’ by SyRI. **Fig. S7.** Expression of alleles in different tissues of R. chinensis ‘CH’. **Fig. S8.** Statistics of ASE gene in different tissues. **Fig. S9.** Phylogenetic tree for ‘CH’ hA and ten other eudicot species contained three outgroups. **Fig. S10.** Gene Ontology (GO) enrichment analysis of expanded gene families in ‘CH’ hA (a) and ‘OB’ (b). **Fig. S11.** Expression of expanded family genes which Gene Ontology associated with terpene synthesis. **Fig. S12.** The differentially expressed genes between noncoloured petals and colouring petals of ‘CH’ hB. **Fig. S13.** Phylogenetic tree analysis of MYB genes from R. chinensis ‘CH’, ‘OB’ and Arabidopsis. **Fig. S14.** The differential genes’ expressions form transcriptome was validated by qRT- PCR. **Fig. S15.** The structural variation of alleles was observed between two haplotypes and two materials by SyRI. **Fig. S16.** The differential expression gene between transgenic roses callus and control roses callus. **Fig. S17.** Promoter sequence of RcMYB114b in R. chinensis ‘OB’ and ‘CH’. **Fig. S18.** GUS staining and GUS enzyme detection. **Fig. S19.** Morphological analysis of ‘OB’ flowers in high temperature treatment and control group. **Fig. S20.** Hierarchical cluster dendrogram showing co-expressed modules identified by weighted gene co-expression network analysis for the rose RNA-seq data. **Fig. S21.** Module-stage association analysis. **Fig. S22.** Gene Ontology (GO) enrichment analysis of four modules genes related to flower organ primordium formation. **Fig. S23.** The DEGs in two comparisons (25ºC ISPS_vs_HBPS and 35ºC ISPS_vs_HBPS). **Fig. S24.** Yeast one-hybrid (Y1H) assay and electrophoretic mobility shift assay (EMSA). **Fig. S25.** Genotype information of RcAP2L. **Fig.S26.** RcAP2L variable spliceosome sequence alignment analysis.

**Additional file 2: Table S1.** Summary of genome sequencing and read out for R. chinensis ‘CH’. **Table S2.** Survey statistic results of R. chinensis ‘CH’. **Table S3.** Summary of Hi-C reads mapping to the R. chinensis. **Table S4.** Result of the evaluation of the R. chinensis genome by BUSCO. **Table S5.** Genome sequence data comparison statistics. **Table S6.** Result of the evaluation of the R. chinensis coding genes by BUSCO. **Table S7.** Gene numbers and features of R. chinensis ‘CH’. **Table S8.** The statistical results of gene function annotation of R. chinensis ‘CH’. **Table S9.** Summary of repeat contents in R. chinensis ‘CH’ genome. **Table S10.** Structural variation between two haplotypes of R. chinensis ‘CH’. **Table S11.** SNP variation between two haplotypes of R. chinensis ‘CH’. **Table S12.** Genes related to flower development in DEGs **Table S13.** List of primers used.

## Data Availability

All data supporting the results of this study are included in the manuscript and its additional files. Genome assembly and annotations were deposited in the NCBI BioProject under accessions PRJNA932466.

## Abbreviations

AP2L: APETALA2-like
ASE: Allele-specific expression
BUSCO: Benchmarking universal single-copy ortholog
CCS: Circular consensus sequencing
CDS: Coding sequences
CHS: Chalcone synthase
DEGs: Differentially expressed genes
DFR: Dihydroflavonol 4-reductase
ERF: Ethylene response factor
F3H: Flavanone 3-hydroxylase
F3’H: Flavonoid 3'-hydroxylase
FLS: Flavonol synthase
GO: Gene ontology
LAI: The long terminal repeat (LTR)-assembly index
LAR: Leucoanthocyanidin reductase
LC–MS: Liquid chromatography-mass spectrometry
LTR-RTs: Long terminal repeat retrotransposons
miR172: MicroRNA172
ORF: Open reading frame
PAL: Phenylalanine ammonia-lyase
qRT‒PCR: Real-time quantitative PCR
RLL1: Red Lettuce Leaves 1
SNP: Single nucleotide polymorphism
SyRI: Synteny and rearrangement identifier
TEs: Transposable elements
TFs: Transcription factors
TPM: Transcript per million
GUS: β-Glucuronidase
WGCNA: Weighted correlation network analysis
