# Supplementary material for: Haplotype-resolved genome assembly of the diploid Rosa chinensis provides insight into the mechanisms underlying key ornamental traits
Source: Mol Hortic. 2024 Apr 16;4:14. doi: 10.1186/s43897-024-00088-1 (PMC11020927; doi:10.1186/s43897-024-00088-1)
Supplement: Supplementary file 1 — Additional file 1: Fig. S1. K-mer analysis for estimating the genome size of R. chinensis ‘CH’ indicating high heterozygosity. Fig. S2. The plot depicts the K-mer spectra of the haplotype assembly. Fig. S3. Hi-C interaction heatmaps of the two haplotypes (hap A and hap B) in R. chinensis ‘CH’. Fig. S4. Sequence depth and GC content for the two haplotypes in R. chinensis ‘CH’. Fig. S5. Dot plots with respect to the ‘OB’ genome reference for the two haplotypes assemblies of ‘CH’ respectively.Fig. S6. Collinearity of genomes from ‘CH’ and ‘OB’ by SyRI. Fig. S7. Expression of alleles in different tissues of R. chinensis ‘CH’. Fig. S8. Statistics of ASE gene in different tissues. Fig. S9. Phylogenetic tree for ‘CH’ hA and ten other eudicot species contained three outgroups. Fig. S10. Gene Ontology (GO) enrichment analysis of expanded gene families in ‘CH’ hA (a) and ‘OB’ (b). Fig. S11. Expression of expanded family genes which Gene Ontology associated with terpene synthesis. Fig. S12. The differentially expressed genes between noncoloured petals and colouring petals of ‘CH’ hB. Fig. S13. Phylogenetic tree analysis of MYB genes from R. chinensis ‘CH’, ‘OB’ and Arabidopsis. Fig. S14. The differential genes’ expressions form transcriptome was validated by qRT- PCR. Fig. S15. The structural variation of alleles was observed between two haplotypes and two materials by SyRI. Fig. S16. The differential expression gene between transgenic roses callus and control roses callus. Fig. S17. Promoter sequence of RcMYB114b in R. chinensis ‘OB’ and ‘CH’. Fig. S18. GUS staining and GUS enzyme detection. Fig. S19. Morphological analysis of ‘OB’ flowers in high temperature treatment and control group. Fig. S20. Hierarchical cluster dendrogram showing co-expressed modules identified by weighted gene co-expression network analysis for the rose RNA-seq data. Fig. S21. Module-stage association analysis. Fig. S22. Gene Ontology (GO) enrichment analysis of four modules genes related to flower or [file 43897_2024_88_MOESM1_ESM.docx]

# Haplotype-resolved genome assembly of the diploid *Rosa chinensis* provides insight into the mechanisms underlying key ornamental traits

Xiaoni Zhang^1,2,3#^, Quanshu Wu^1#^, Lan Lan^2,3,4^, Dan Peng^2,3^, Huilin Guan^1^, Kaiqing Luo^2,3^, Manzhu Bao^1^, Mohammed Bendahmane^1,5^*****, Xiaopeng Fu^1^*****, Zhiqiang Wu^2,3^*****

1. National Key Laboratory for Germplasm Innovation & Utilization of Horticultural Crops, College of Horticulture and Forestry Sciences, Huazhong Agricultural University, Wuhan, 430070, China.
2. Shenzhen Branch, Guangdong Laboratory of Lingnan Modern Agriculture, Key Laboratory of Synthetic Biology, Laboratory of the Ministry of Agriculture and Rural Affairs, Agricultural Genomics Institute at Shenzhen, Chinese Academy of Agricultural Sciences, Shenzhen, 518120, China.
3. Kunpeng Institute of Modern Agriculture at Foshan, Foshan, 528200, China.
4. College of Science, Health, Engineering and Education, Murdoch University, 6150, Western Australia, Murdoch, 90 South Street, Australia.
5. Laboratoire Reproduction et Development des Plantes, INRA-CNRS-Lyon1-ENS, Ecole Normale Supérieure de Lyon, Lyon 520074, France.

# Xiaoni Zhang and Quanshu Wu contributed equally to this work.

*Correspondence:

[wuzhiqiang@caas.cn](mailto:wuzhiqiang@caas.cn); Tel.: +86 135-3040-6763

[fuxiaopeng@mail.hzau.edu.cn](mailto:fuxiaopeng@mail.hzau.edu.cn); Tel.: +86-159-2625-8658;

[mohammed.bendahmane@ens-lyon.fr](mailto:mohammed.bendahmane@ens-lyon.fr);

**Supplementary Notes**

**Plant materials**

To analyze the effect of high-temperature treatment on rose flower development, sixty individual ‘CH’ plants were pruned and then randomly placed into two incubators with two different parameter settings: (1) 16 / 8 h day/night at 35 °C/30 °C and (2) 16 / 8 h day/night at 25 °C/20 °C. Thirty individual ‘CH’ plants under treatment were randomly divided into three groups, and each group contained 10 ‘CH’ plants for sample collection as one biological. After 50 d, the four different stages of flower buds: the vegetative meristem stage (DBO); the initiation stages of petals/petal-like structures and stamens/stamen-like structures (ISPS); the stage in which the hypanthium starts to sink below the perianth and stamens (HBPS); and the stage of flower buds with young noncoloured petals (FBGP), were observed individually and were collected under a microscope (SOPTOP SZN, Yuyao, China) in three biological replicates for RNA-seq (Dubois et al., 2010; Dubois et al., 2012; Han et al., 2017).The ISPS consists two closely related stages: the initial (ISPS1) and development (ISPS2) stage of petal/stamen primordia. Because its development process is overlapping, so as one stage. Each biological replicate contained at least 50 flower buds from 10 plants.

**WGCNA**

The co-expression networks were constructed using the WGCNA package (Langfelder and Horvath 2008) to identify relationships between traits and stages. The parameters used in the construction of WGCNA were weighted network, unsigned, hierarchal clustering tree, dynamic hybrid tree cut algorithm, power 8.

**Phylogenetic and alignment analysis of candidate MYB genes**

Phylogenetic analysis was performed using *Arabidopsis* and the candidate *MYB* genes in rose. Protein sequences were obtained from Tair (<https://www.arabidopsis.org/>) and previous reports (Stracke et al. 2001). Phylogenetic tree was constructed using MEGA v6.0 (Tamura et al., 2013) by the neighbor joining (NJ) method with 1000 iterations for the bootstrap values.

**Promoter and CDS sequences comparison of *RcMYB114b***

The promotors of *RcMYB114b* (1500bp upstream of start codon) were cloned from ‘OB’ and ‘CH’ leaves DNA and fused into pCE2 TA/Blunt-Zero Vector (Vazyme, Nanjing, China). The CDS of *RcMYB114b* was amplified from ‘OB’ and ‘CH’ cDNA using corresponding primer sequences (Table S13) and cloned into the pCE2 TA/Blunt-Zero Vector (Vazyme, Nanjing, China). The Nucleotide sequences were check by sequencing at Sangon (Wuhan, China) and aligned with Snap Gene v6.0.2 program. DNAMAN software was used to perform the figures.

**Transient gene expression in rose petals**

*RcMYB114bpro^short^* and *RcMYB114bpro^long^* were constructed on DX2181G, respectively. The 35S core promoter sequence was constructed on DX2181G vector as a positive control, and the empty vector was used as a negative control. The constructed vectors were respectively transformed into *Agrobacterium* GV3101, and the petals of ‘OB’ were transiently infected according to the method of Zeinipour et al (2018).

**GUS (β-glucuronidase) staining and fluorometric assays**

Infected petals were incubated at 22 ºC for 1 d in the dark, and samples after 3 d of incubation at 22 ºC were subjected to GUS staining or flash frozen in liquid nitrogen (Yasmin and Debener, 2010) for GUS fluorescence analysis. For histochemical staining of GUS, petals were soaked in staining solution using GUS staining kit (Coolaber, SL7160) and overnight incubation at 37ºC. Stained samples were bleached with 75 % (v/v) ethanol and observed with microscopes (COIC ZSA0745T, Chongqing, China). For fluorometric assays, 100 mg of sample was used for quantitative detection according to GUS reporter gene Quantitative detection kit (Coolaber, SL7161). Protein concentration was determined by Bradford Protein Content Assay Kit (Boxbio, AKPR015). Fluorescence was determined using a spectrophotofluorometer (Shimadzu, RF-5301PC, Japan). one–way ANOVA along with the least significant difference test and graphing were performed using GraphPad Prism 9. *P* < 0.05 was considered significant.

**Yeast one-hybrid assay**

For Yeast one-hybrid assay, the promoter fragments of *RcAG1* (1,445bp upstream of start codon) were inserted into the pHis2 vector to construct *RcAG1pro-pHis2*. The ORF of *RcSVP1* was inserted into the pGADT7 (AD) vector to construct *RcSVP1-AD* (Table S16). The recombinant constructs were co-transformed into yeast strain Y187. Transformants were cultured in SD/-Leu-Trp (-L/-T) and SD/-His-Leu-Trp (-H/-L/-T) media with 8mM 3-amino-1,2,4-triazole (3AT) for 3 d in 30 °C.

**Electrophoretic mobility shift assay**

The full-length CDS of *RcSVP1* was cloned into the pHGWA vector with a His tag (Table S17). The vectors were transformed in *Escherichia coli* strain Rosetta (DE3). The recombinant proteins were induced at 16°C and purified in its native form using Ni-NTA agarose (Transgen) according to the manufacturer's instructions. The *RcAG1pro* probe (40 bp) was synthesized and labeled with biotin at the 5′terminals by Sangon Biotech (Beijing, China), whereas the unlabeled mutated and WT probes were used as a competitor. Electrophoretic mobility shift assay (EMSA) and Visualization were carried out using a LightShift Chemiluminescent EMSA Kit (Thermo Fisher Scientific) and Chemiluminescence Nucleic Acid Detection Module (Thermo Fisher Scientific), separately, according to the manufacturer's protocols.

**Reference:**

Dubois, A., Raymond, O., Maene, M., Baudino, S., Langlade, N.B., Boltz, V., Vergne, P., and Bendahmane, M. Tinkering with the C-Function: A Molecular Frame for the Selection of Double Flowers in Cultivated Roses. Plos One. 2010, 5: e9288.

Dubois, A., Carrere, S., Raymond, O., Pouvreau, B., Cottret, L., Roccia, A., Onesto, J.P., Sakr, S., Atanassova, R., Baudino, S., et al. Transcriptome database resource and gene expression atlas for the rose. BMC genom. 2012, 13:638.

Han, Y., Wan, H., Cheng, T., Wang, J., Yang, W., Pan, H., and Zhang, Q. Comparative RNA-seq analysis of transcriptome dynamics during petal development in *Rosa chinensis*. Sci Rep. 2017, 7:43382.

Langfelder, P. and Horvath, S. WGCNA: an R package for weighted correlation network analysis. BMC Bioinformatics. 2008, 9:559.

Stracke, R., Werber, M. and Weisshaar, B. The R2R3-MYB gene family in *Arabidopsis thaliana*. Curr Opin Plant Biol. 2001, 4(5):447-56.

Tamura, K., Stecher, G., Peterson, D., Filipski, A., and Kumar, S. Mega6: molecular evolutionary genetics analysis version 6.0. Molecular Biology & Evolution. 2013, 30(12):2725-2729.

Yasmin, A. and Debener, T. Transient gene expression in rose petals via Agrobacterium infiltration. Plant Cell Tiss Organ Cult. 2010, 102:245–250.

Zeinipour, M., Azadi, P., Majd, A., Kermani, M.J., Irian, S., Hosseini, S.M. and Mii, M. Agroinfiltration: a rapid and reliable method to select suitable rose cultivars for blue flower production. Physiol Mol Biol Plants. 2018, 24(3):503-511.

**Supplementary Tables**

Table S1. Summary of genome sequencing and read out for *R. chinensis* ‘CH’.

Table S2. Survey statistic results of *R. chinensis* ‘CH’.

Table S3. Summary of Hi-C reads mapping to the *R. chinensis.*

Table S4. Result of the evalutation of the *R. chinensis* genome by BUSCO.

Table S5. Genome sequence data comparison statistics.

Table S6. Result of the evalutation of the *R. chinensis* coding genes by BUSCO.

Table S7. Gene numbers and features of *R. chinensis* ‘CH’.

Table S8. The statistical results of gene function annotation of *R. chinensis* ‘CH’.

Table S9. Summary of Repeat contents in *R. chinensis* ‘CH’ genome.

Table S10. Structural variation between two haplotypes of *R. chinensis* ‘CH’.

Table S11. SNP variation between two haplotypes of *R. chinensis* ‘CH’.

Table S12. Genes related to flower development in DEGs.

Table S13. Primers for CDS amplification of *RcMYB114b* gene.

Table S14. Primers for qRT-PCR in rose.

Table S15. Primers for dual luciferase assays of *RcSVP1* and *RcAG1* genes.

Table S16. Primers for yeast one-hybrid assays.

Table S17. Primers for electrophoretic mobility shift assay.

**Supplementary Figures**

Fig. S1. K-mer analysis for estimating the genome size of *R. chinensis* ‘CH’ indicating high heterozygosity.

Fig. S2. The plot depicts the K-mer spectra of the haplotype assembly.

Fig. S3. Hi-C interaction heatmaps of the two haplotypes (hap A and hap B) in *R. chinensis* ‘CH’.

Fig. S4. Sequence depth and GC content for the two haplotypes in *R. chinensis* ‘CH’.

Fig. S5. Dot plots with respect to the ‘OB’ genome reference for the two haplotypes assemblies of ‘CH’ respectively.

Fig. S6. Collinearity of genomes from ‘CH’ and ‘OB’ by SyRI.

Fig. S7. Expression of alleles in different tissues of *R. chinensis* ‘CH’.

Fig. S8. Statistics of ASE gene in different tissues.

Fig. S9. Phylogenetic tree for ‘CH’ hA and ten other eudicot species contained three outgroups.

Fig. S10. Gene Ontology (GO) enrichment analysis of expanded gene families in ‘CH’ hA (a) and ‘OB’ (b).

Fig. S11. Expression of expanded family genes which Gene Ontology associated with terpene synthesis.

Fig. S12. The differentially expressed genes between noncoloured petals and colouring petals of ‘CH’ hB.

Fig. S13. Phylogenetic tree analysis of *MYB* genes from *R. chinensis* ‘CH’, ‘OB’ and *Arabidopsis*.

Fig. S14. The differential genes’ expressions form transcriptome was validated by qRT- PCR.

Fig. S15. The structural variation of alleles was observed between two haplotypes and two materials by SyRI.

Fig. S16. The differential expression gene between transgenic roses callus and control roses callus.

Fig. S17. Promoter sequence of *RcMYB114b* in *R. chinensis* ‘OB’ and ‘CH’.

Fig. S18. GUS staining and GUS enzyme detection.

Fig. S19. Morphological analysis of ‘OB’ flowers in high temperature treatment and control group.

Fig. S20. Hierarchical cluster dendrogram showing co-expressed modules identified by weighted gene co-expression network analysis for the rose RNA-seq data.

Fig. S21. Module-stage association analysis.

Fig. S22. Gene Ontology (GO) enrichment analysis of four modules genes related to flower organ primordium formation.

Fig. S23. The DEGs in two comparisons (25ºC ISPS_vs_HBPS and 35ºC ISPS_vs_HBPS).

Fig. S24. Yeast one-hybrid (Y1H) assay and electrophoretic mobility shift assay (EMSA).

Fig. S25. Genotype information of *RcAP2L*.

Fig. S26. *RcAP2L* variable spliceosome sequence alignment analysis.


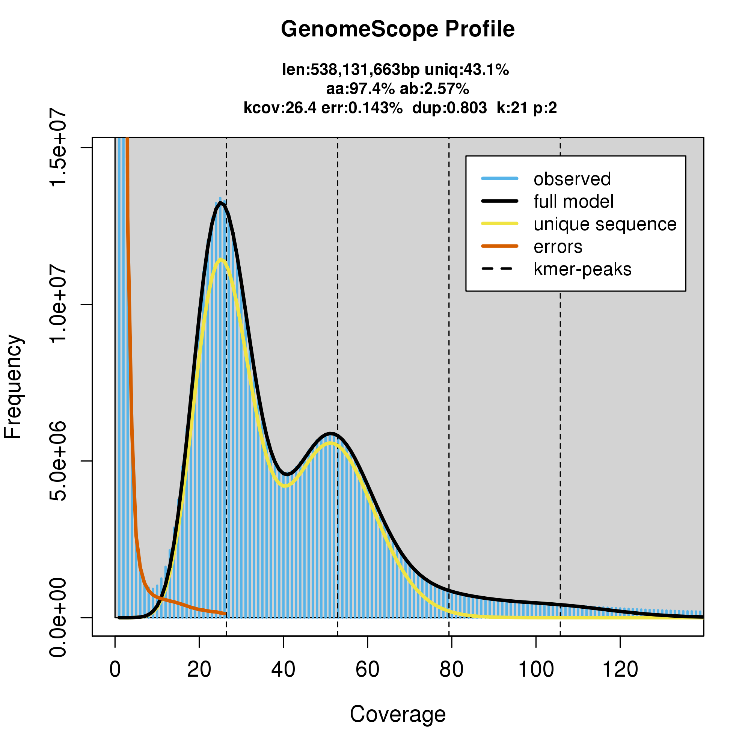


Fig. S1. K-mer analysis for estimating the genome size of *R. chinensis* ‘CH’ indicating high heterozygosity.


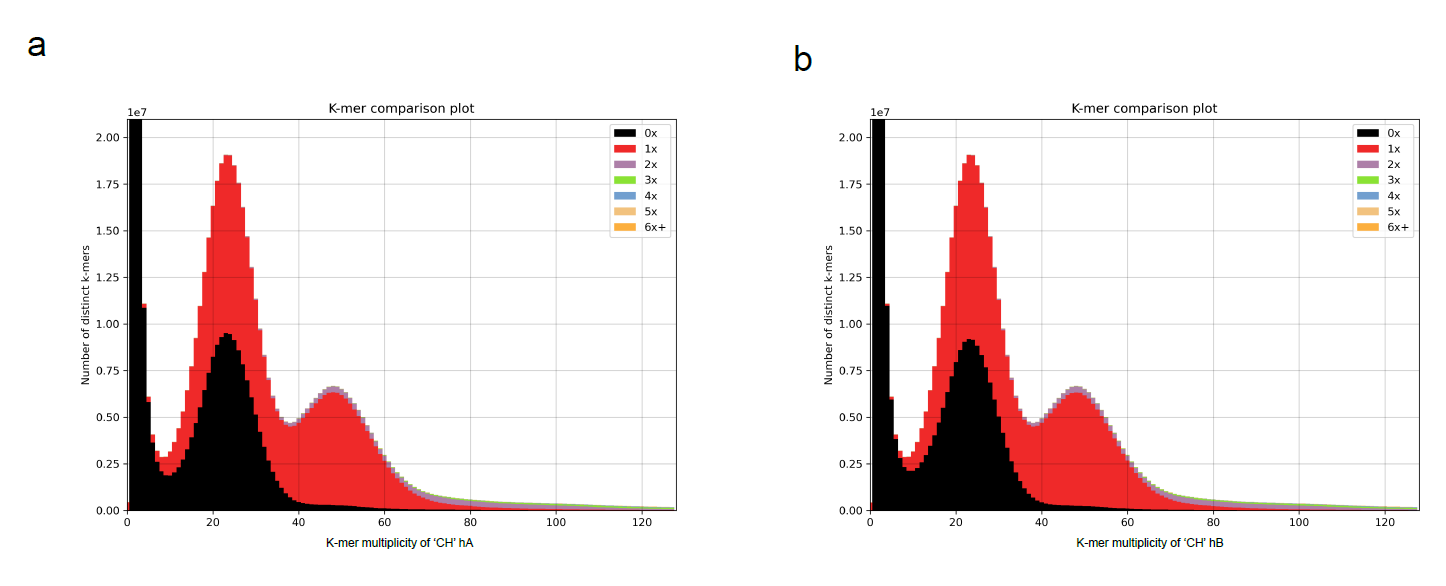


Fig. S2. The plot depicts the K-mer spectra of the haplotype assembly. The x-axis indicates the multiplicity of distinct k-mers of raw HiFi reads. The colors represent the times the k-mers were present in the genome assembly.


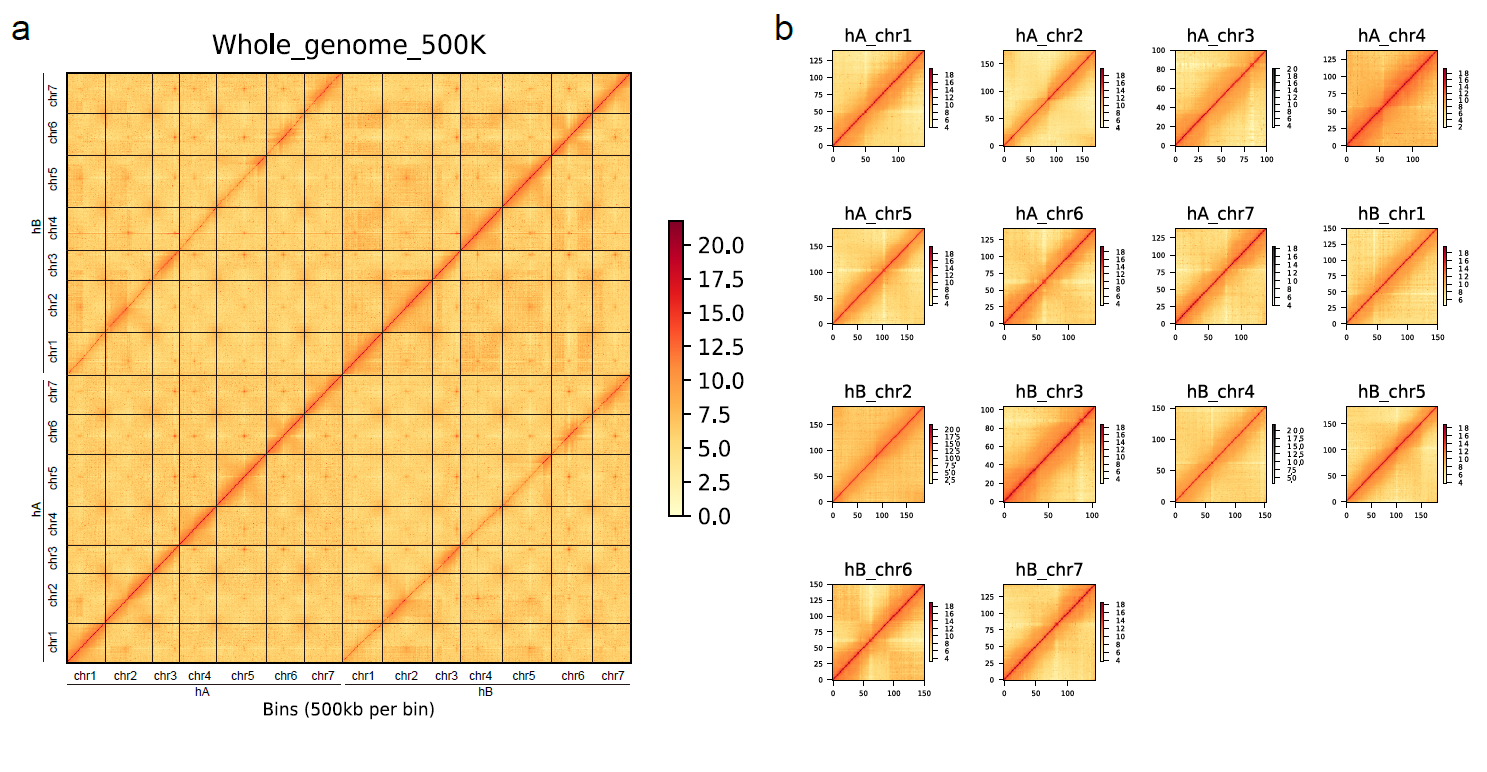


Fig. S3. Hi-C interaction heatmaps of the all chromosomes (a) and each chromosomes (b) in *R. chinensis* ‘CH’. The x-axis and y-axis indicates all chromosomes in 500kb per bin in photo of (a). The x-axis and y-axis means the number of bins in photo of (b).


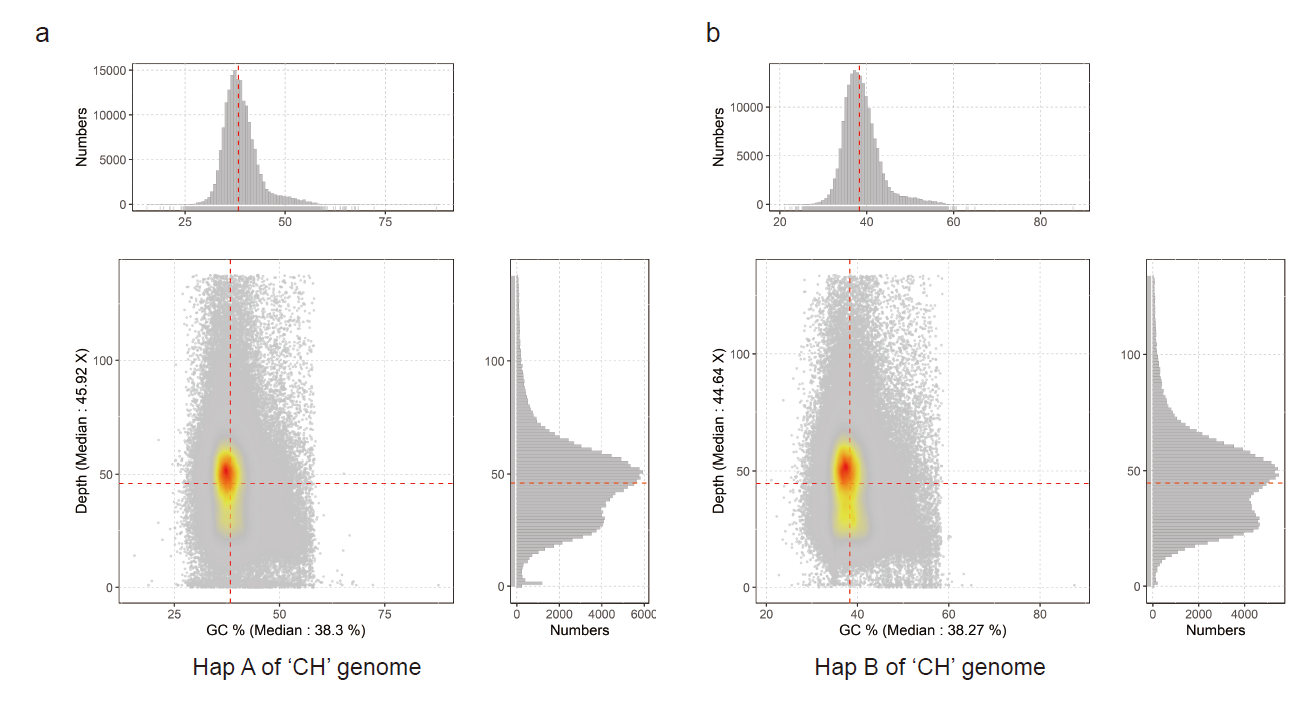


Fig. S4. Sequence depth and GC content for the two haplotypes in *R. chinensis* ‘CH’.


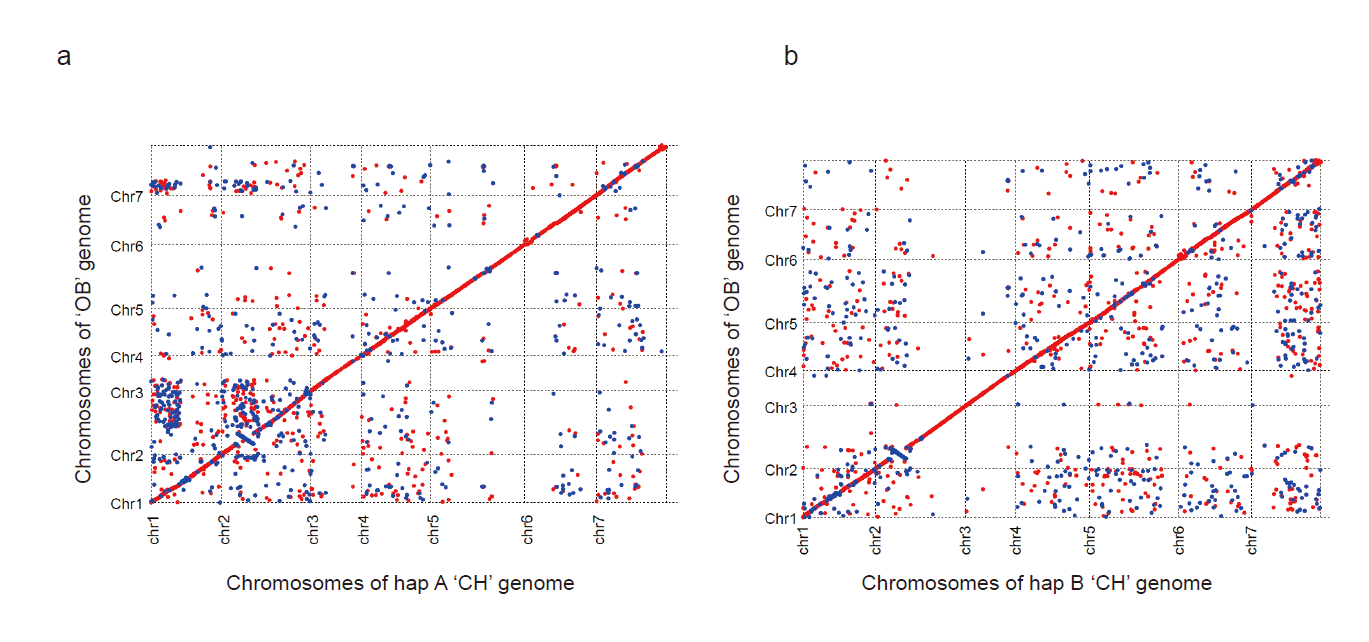


Fig. S5. Dot plots with respect to the ‘OB’ genome for the two haplotypes assemblies of ‘CH’, respectively. The ‘OB’ genome was as query genome. Hap A or hap B was as the reference genome.


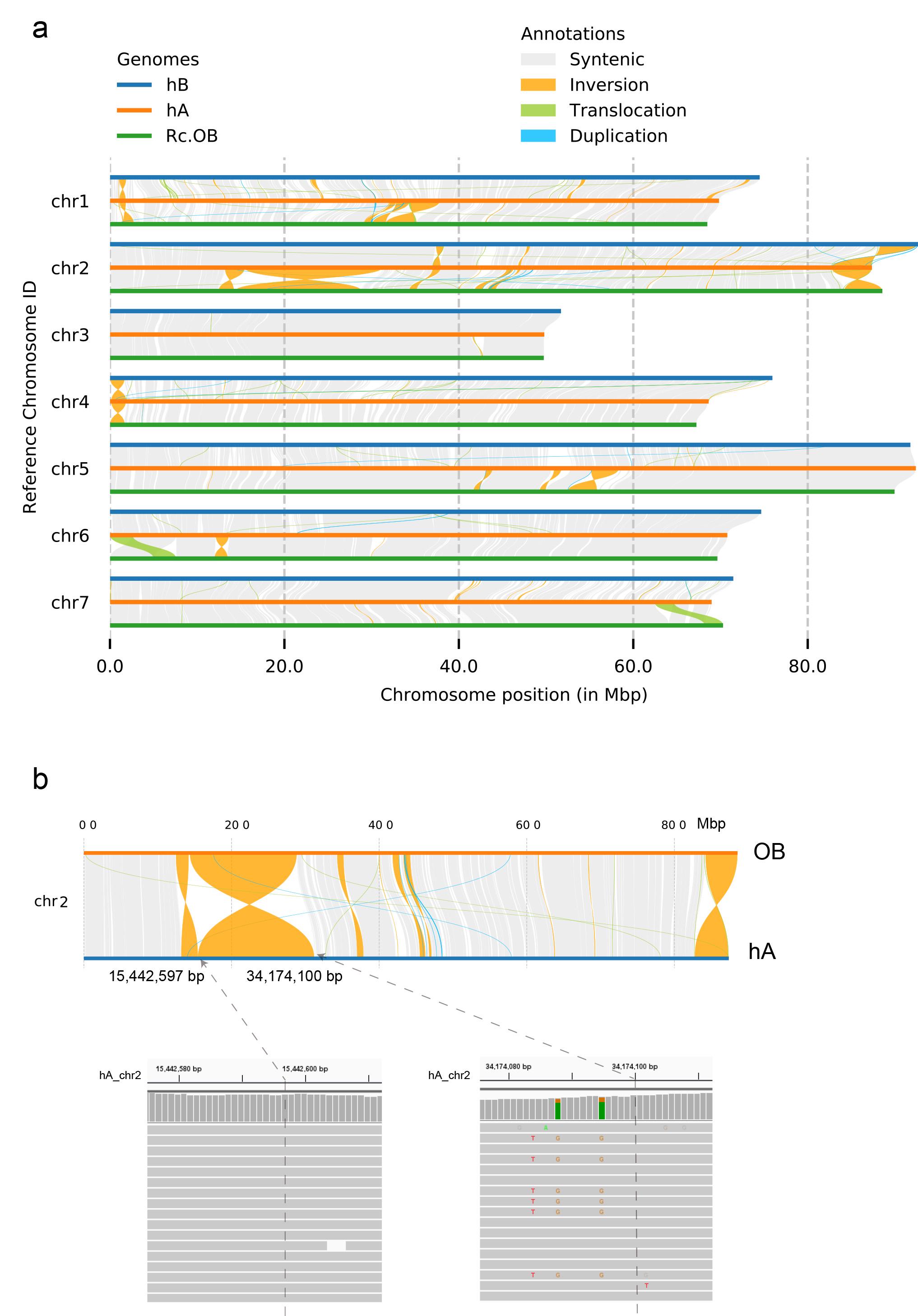


Fig. S6. Collinearity of genomes from ‘CH’ and ‘OB’ by SyRI. a. Collinearity of genome between the two haplotypes (hap A and hap B) of ‘CH’ and the ‘OB’ (RcHm) genome. b. The HiFi reads mapping of inverted region on chromosome 2. There were several hifi reads across both ends of the inversion, indicating that inversion was supported by hifi data. The gray boxes represent hifi reads. The sequence is represented by colored bars, with adenine (A) in green, cytosine (C) in blue, guanine (G) in yellow, and thymine (T) in red.


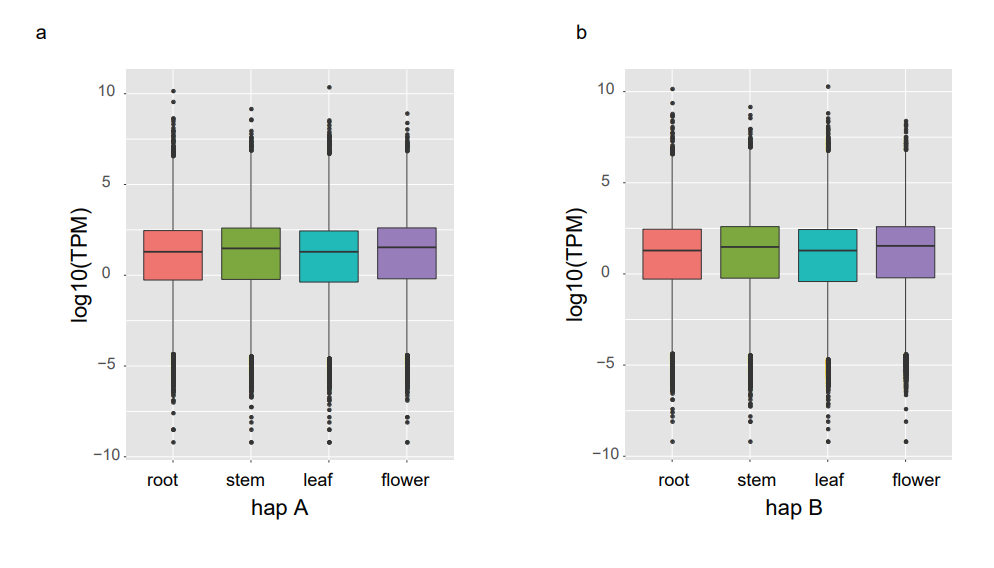
Fig S7. Expression of alleles in different tissues of *R. chinensis* ‘CH’.


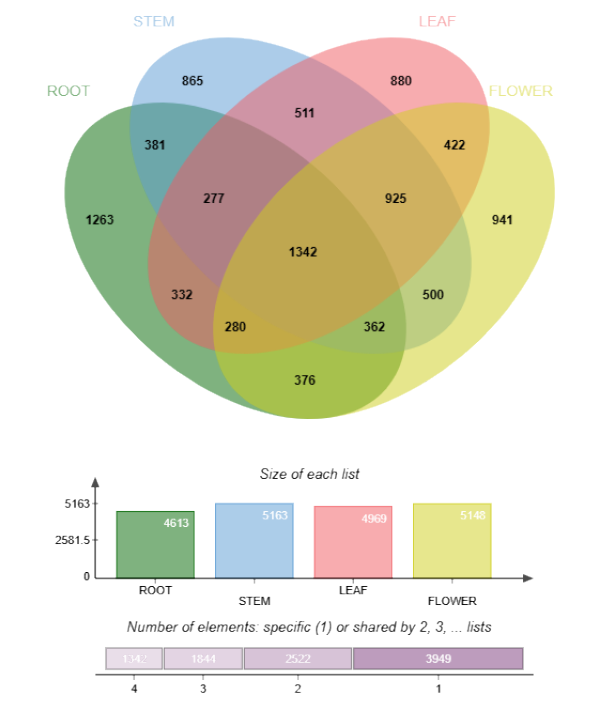


Fig. S8. Statistics of ASE gene in different tissues. Green represents allele-specific expression (ASE) in the root. Blue represents ASE in the stem. Pink represents ASE in the leaf. yellow represents ASE in the flower. Venn diagram of differentially expressed genes in different tissues.


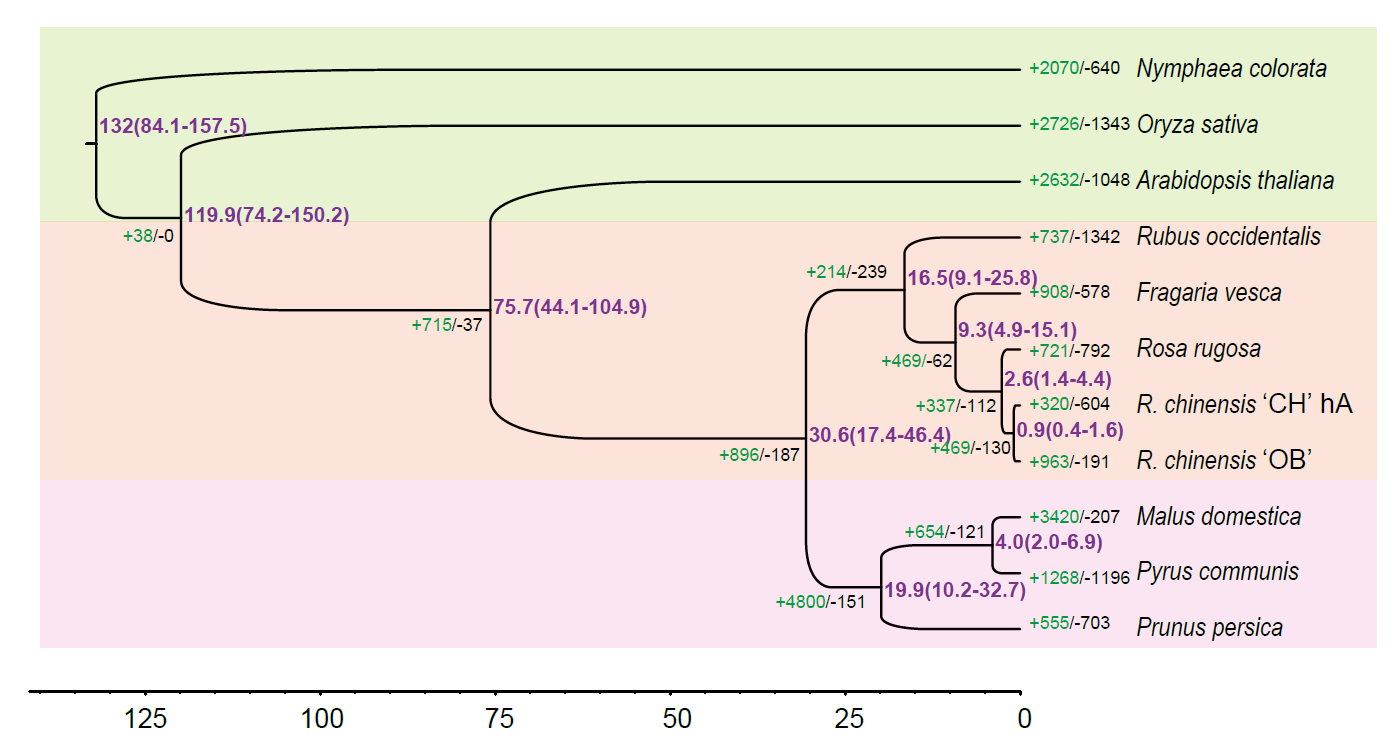


Fig. S9. Phylogenetic tree for ‘CH’ hA and ten other eudicot species contained three outgroups. Gene family expansions are indicated in green, and contractions in black; The estimated divergence time (million years ago, MYA) is indicated at each node; numbers in brackets are the 95% confidence intervals (each center is defined as mean value).


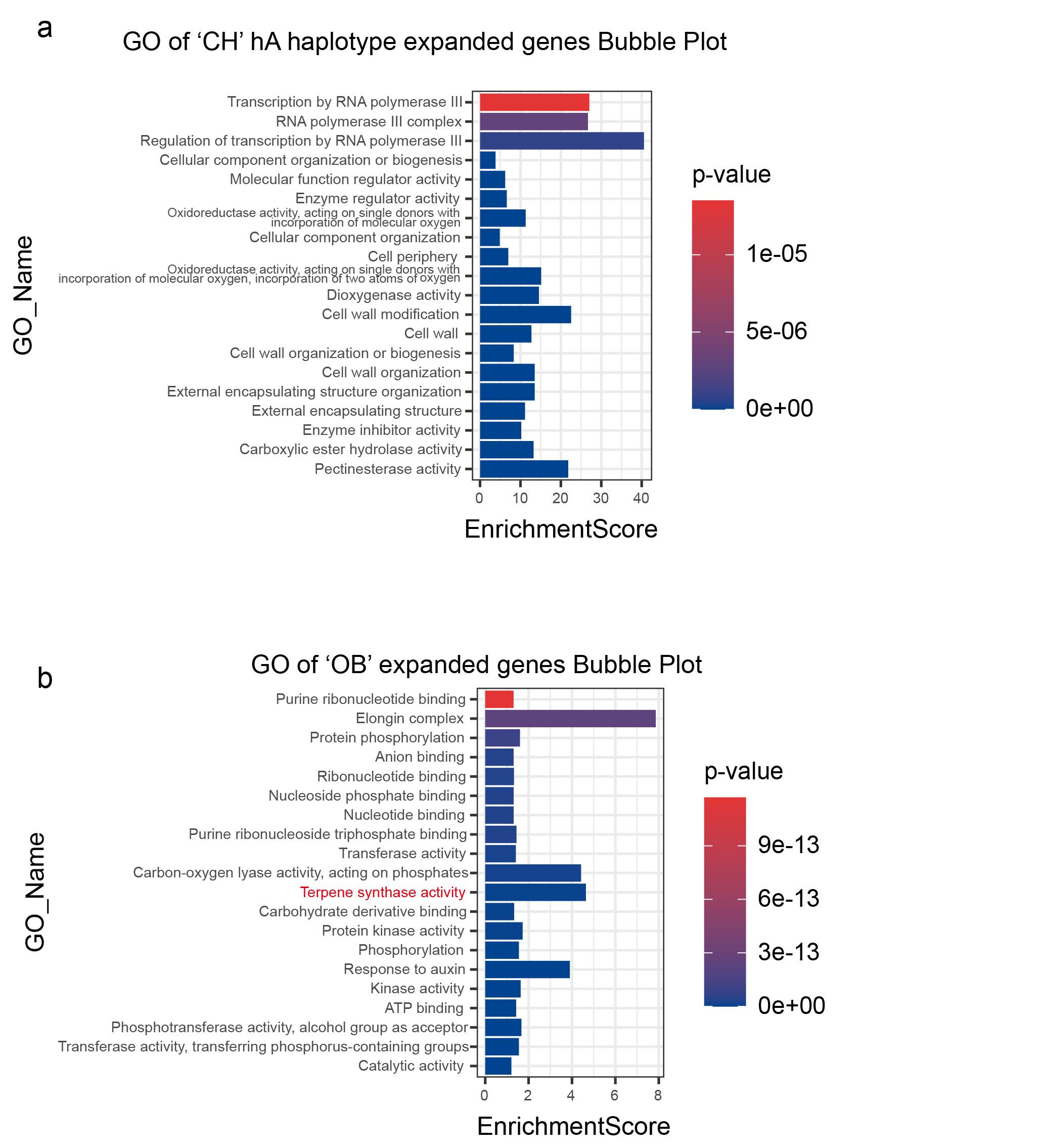


Fig. S10. Gene Ontology (GO) enrichment analysis of expanded gene families in ‘CH’ hA (a) and ‘OB’(b).


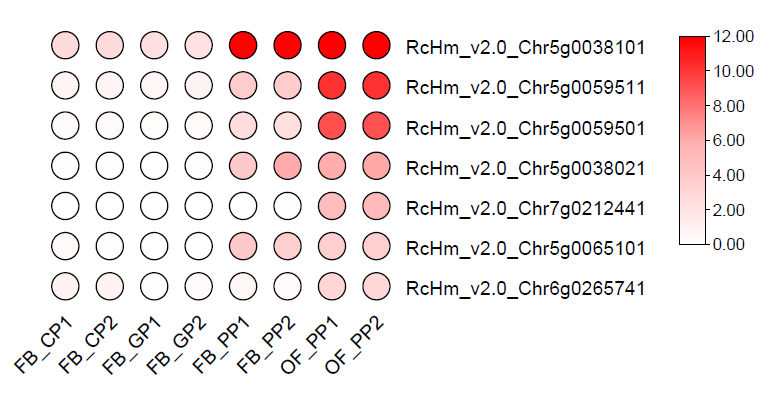


Fig. S11. Expression of expanded family genes which Gene Ontology associated with terpene synthesis. The result shown in the diagram is log_2_ (FPKM). The four stages of petal development of ‘OB’: FB_GP, green petals in the flower bud; FB_CP, colour changing petals in the flower bud; FB_PP, pink petals in the flower bud; OF_PP, pink petals of the open flower.


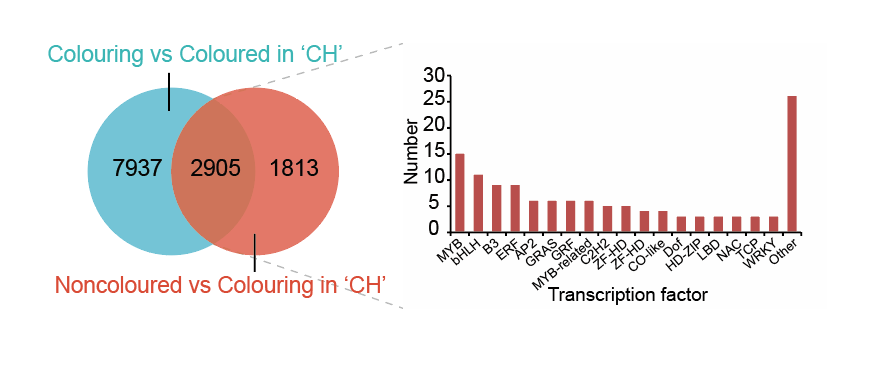
Fig. S12. The differentially expressed genes between noncoloured petals and colouring petals of ‘CH’ hB.


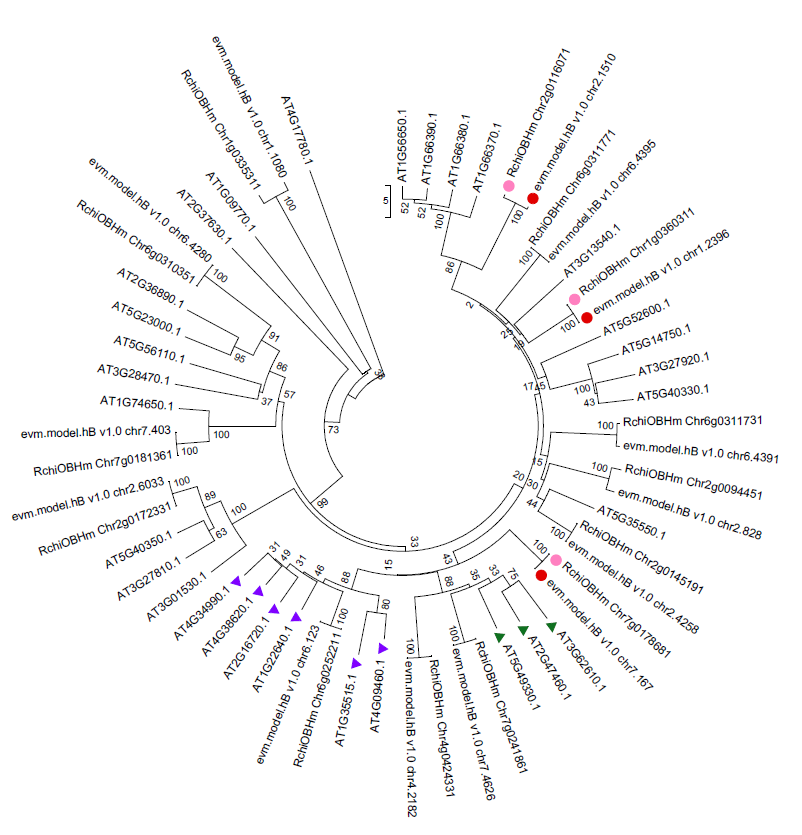


Fig. S13. Phylogenetic tree analysis of MYB genes from *R. chinensis* ‘CH’, ‘OB’ and *Arabidopsis*. Red circles represent MYB candidate genes in ‘CH’, pink circles represent orthologs of candidate genes in ‘OB’, purple and green triangles represent members of the *Arabidopsis* S4 subfamily, and S7 subfamily, separately.


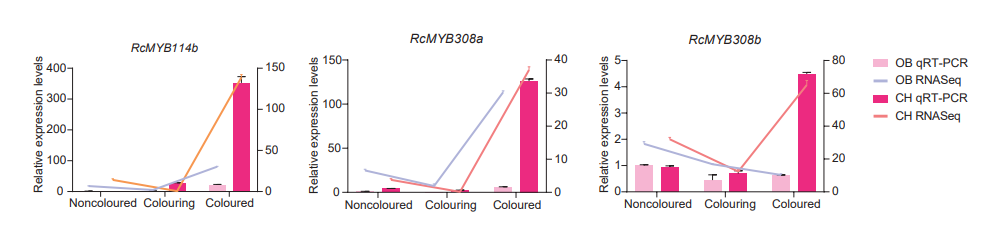
Fig. S14. The differential genes expressions form transcriptome was validated by qRT- PCR.


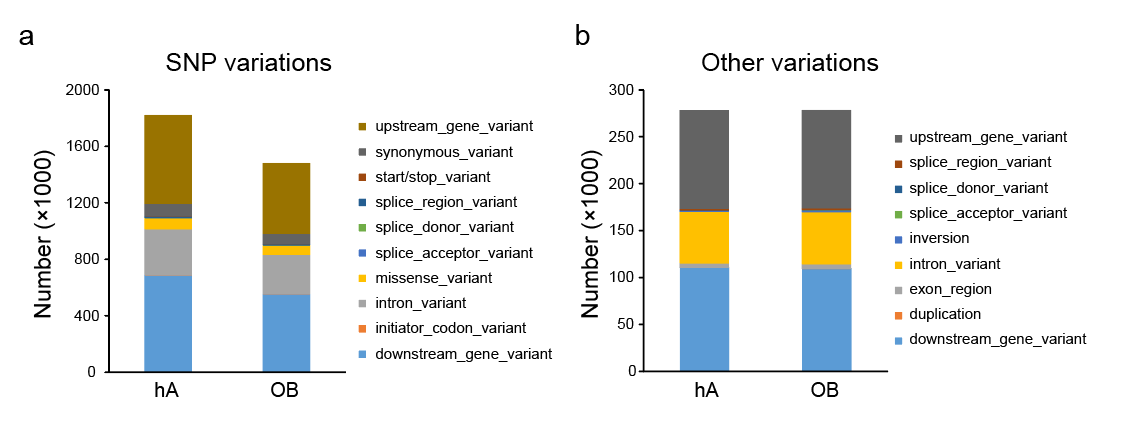


Fig. S15. The structural variation of alleles was observed between two haplotypes and two materials by SyRI. The hB genome of ‘CH’ was selected as reference.


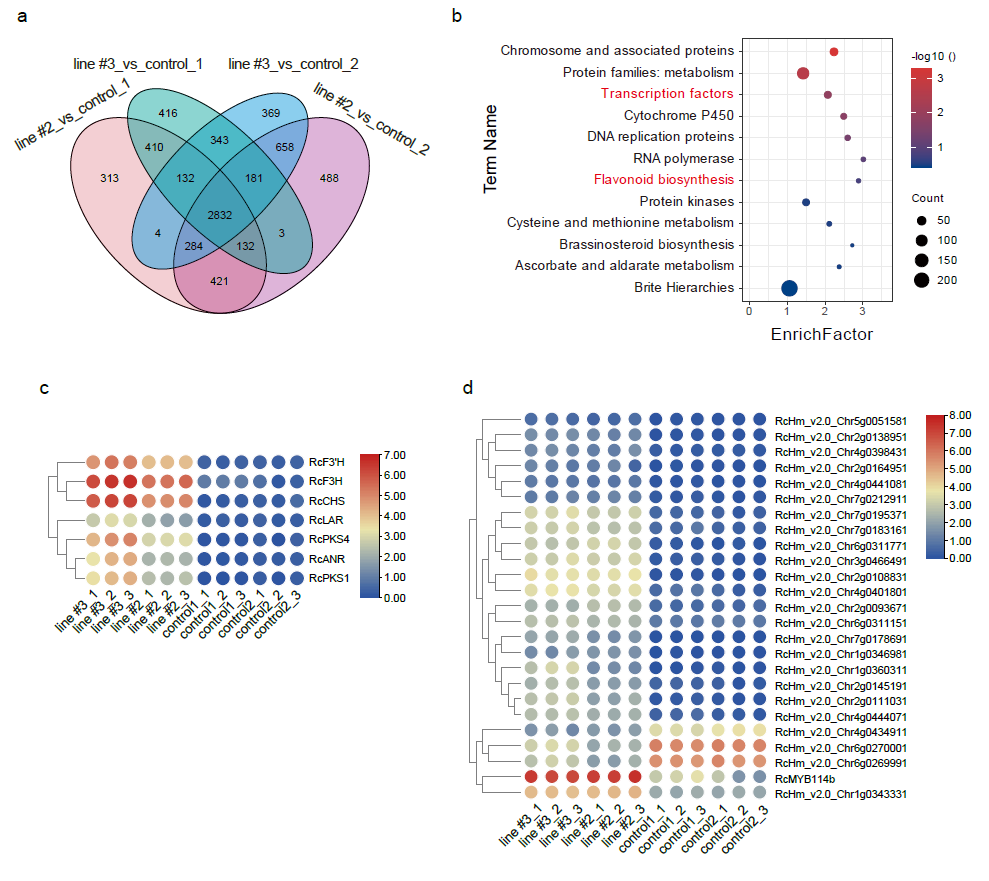


Fig. S16. The differential expression gene between transgenic roses callus and control roses callus. a. Venn diagram of differentially expressed genes. b. The KEGG enrichment of differentially expressed genes. Genes related to flavonoid biosynthesis (c) and transcription factors (d).


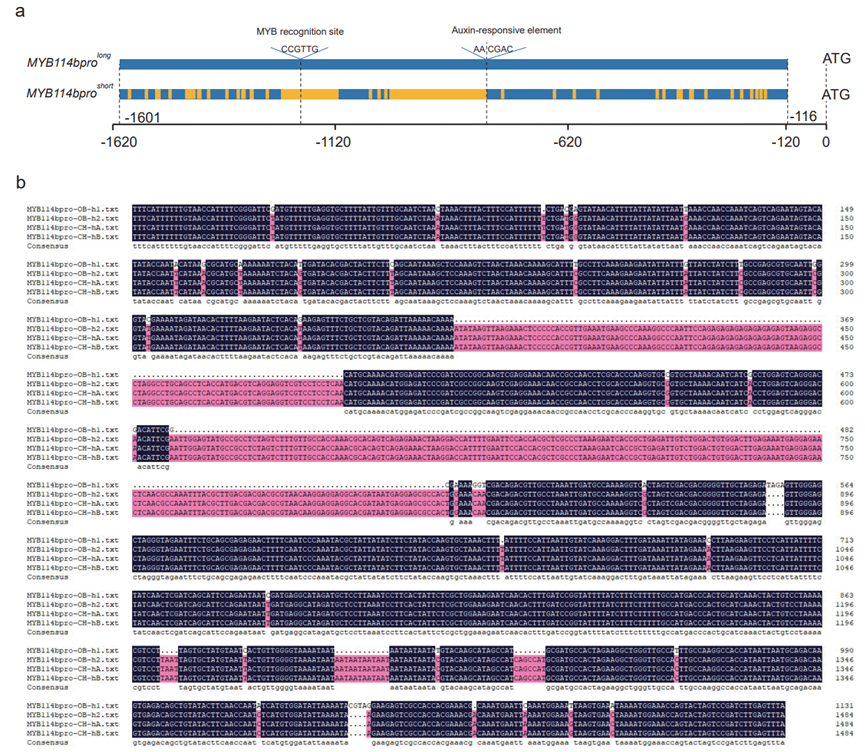


Fig. S17. Promoter sequence of *RcMYB114b* in *R. chinensis* ‘OB’ and ‘CH’. a. Schematic representation of alignment of promoter sequence of *RcMYB114b* in *R. chinensis* ‘OB’ and ‘CH’. The blue squares represent the same sequences, and the yellow regions represent the regions with missing bases or differences in bases. The dotted line indicates the position in the sequence. b. Alignment of promoter sequence of *RcMYB114b* in *R. chinensis* ‘OB’ and ‘CH’.


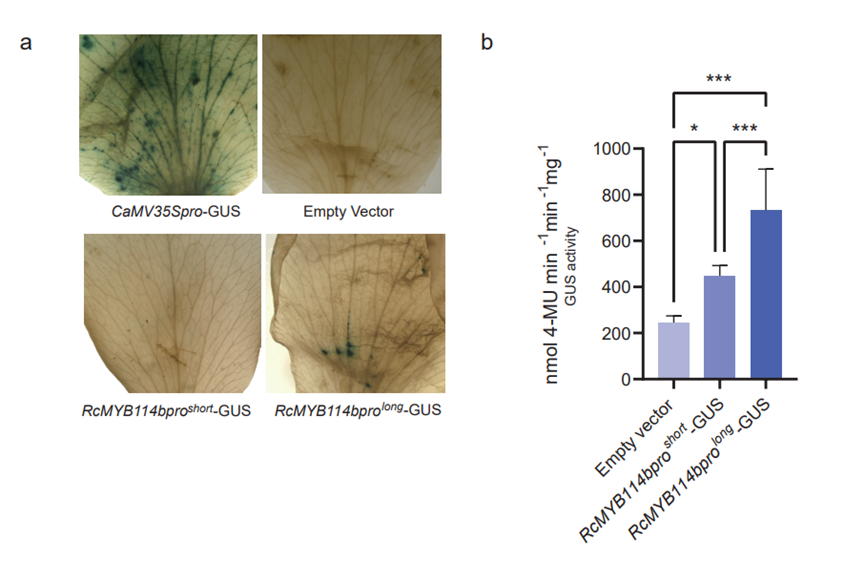


Fig. S18. GUS staining and GUS enzyme detection. a. Transient expression assay in rose ‘OB’ petals and GUS staining assay. b. Fluorometric assays of GUS. The bars indicate the SD. Stars mean significant differences (*** represents *P* < 0.001, ** represents *P* < 0.01, * represents *P* < 0.05) analyzed by one–way ANOVA.


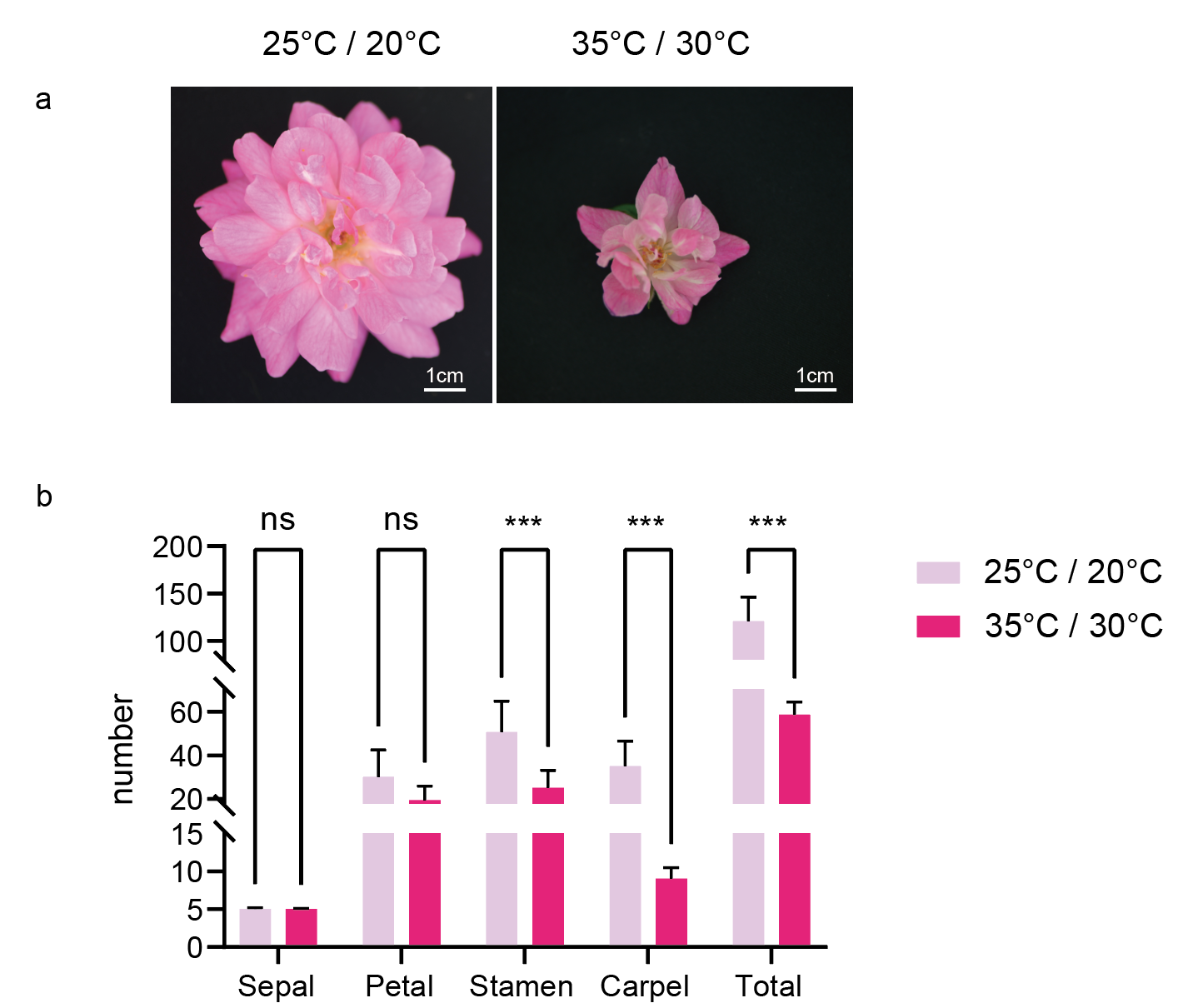


Fig. S19. Morphological analysis of ‘OB’ flowers in high temperature treatment and control group. a. ‘OB’ flower in the high temperature treatment and control group. b. Statistical analysis of the number of ‘OB’ flowering organs in high temperature treatment and control group. Stars mean significant differences (*** represents *P* < 0.001) analyzed by two–way ANOVA.


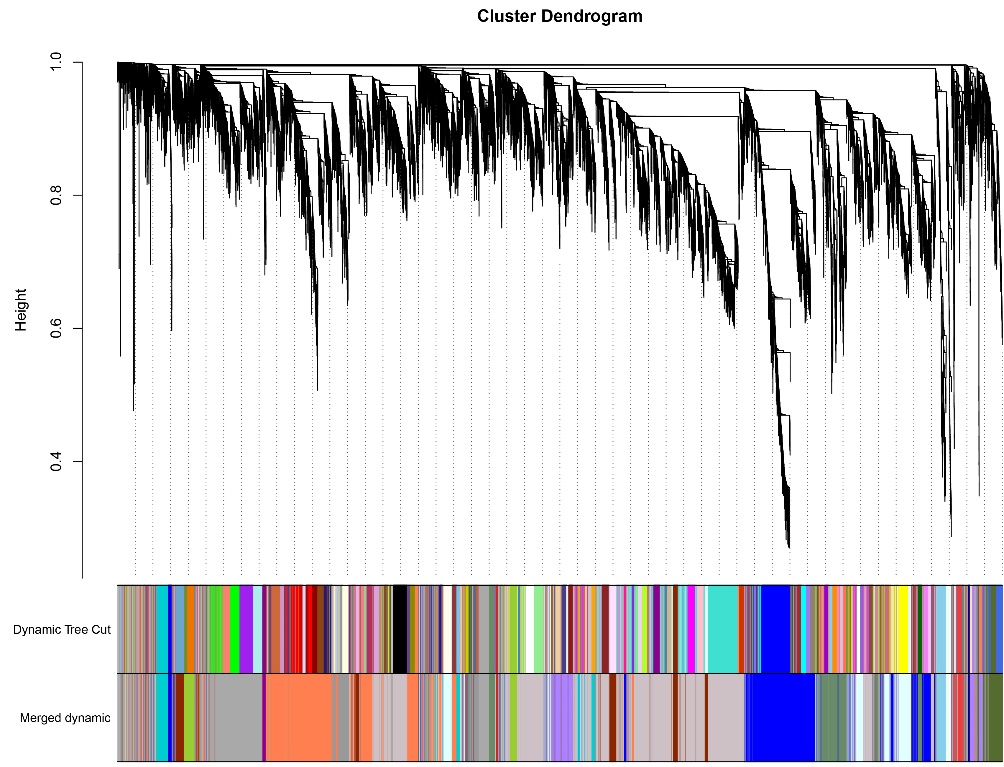


Fig. S20. Hierarchical cluster dendrogram showing co-expressed modules identified by weighted gene co-expression network analysis for the rose RNA-seq data. Each leaf on the tree represents one gene.


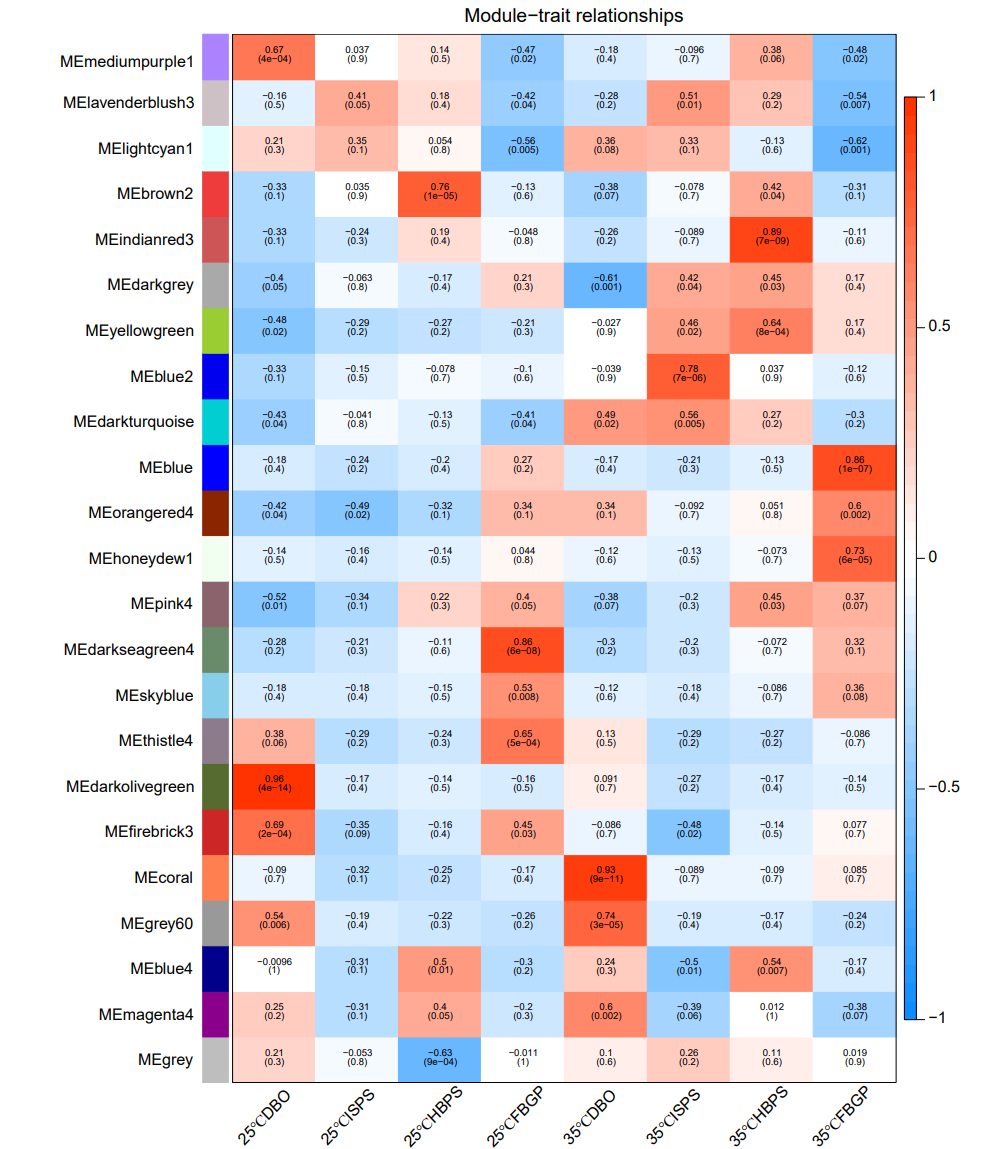


Fig. S21. Module-stage association analysis. Each column corresponds to a specific stage. Red and blue indicate positive and negative correlation, respectively. The vegetative meristem stage (DBO); the initiation stages of petals/petal-like structures and stamens/stamen-like structures (ISPS); the stage in which the hypanthium starts to sink below the perianth and stamens (HBPS); and the stage of flower buds with young noncoloured petals (FBGP).


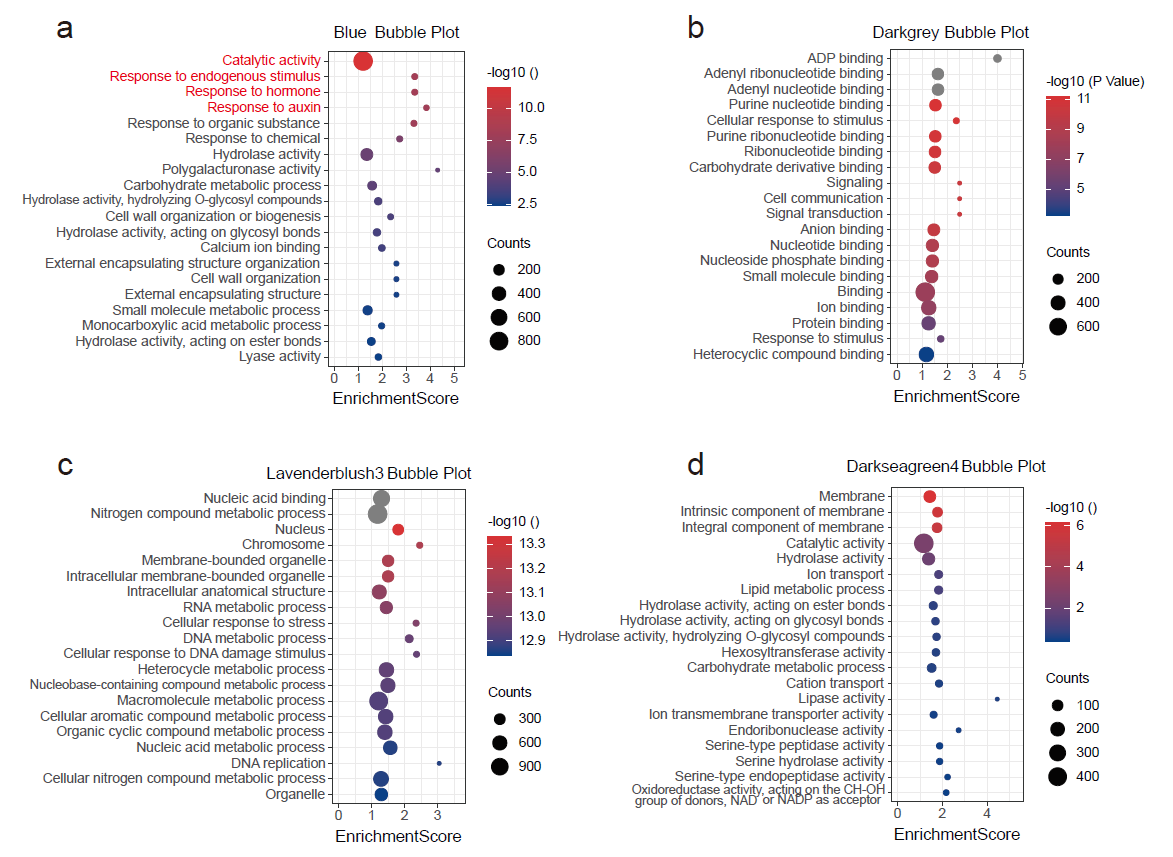


Fig. S22. Gene Ontology (GO) enrichment analysis of four modules genes related to flower organ primordium formation. The four modules contained Blue module (a), Darkgrey module (b), Lavenderblush module (c) and Darkseagreen4 module (e). Enrichment values are indicated by colour, as shown in the colour scale. The red letters represent those genes that may affect petal size.


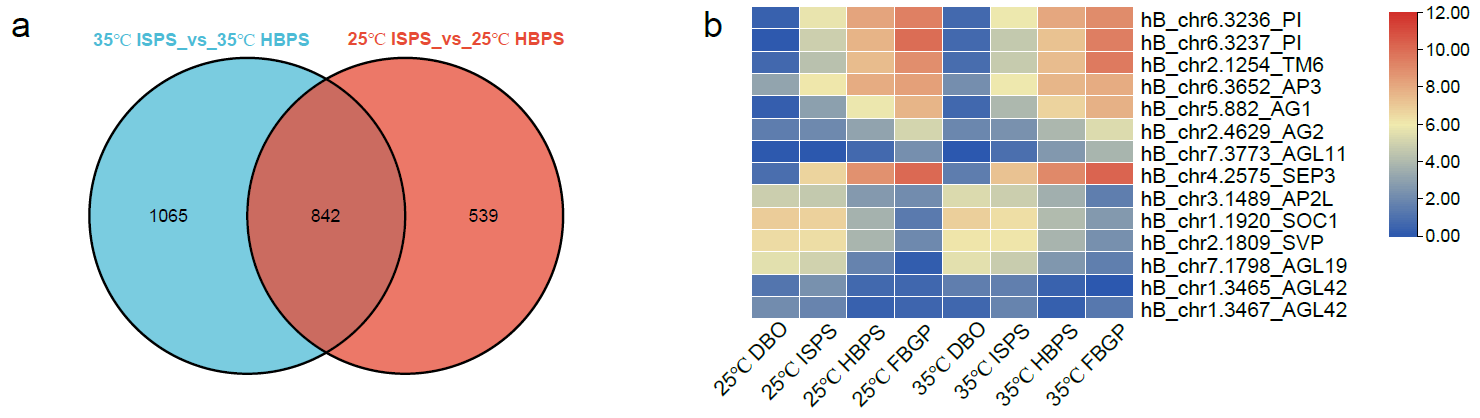


Fig. S23. The DEGs in two comparisons (25ºC ISPS_vs_HBPS and 35ºC ISPS_vs_HBPS). a.Venn diagram indicates the number of DEGs in two comparisons (25ºC ISPS_vs_HBPS and 35ºC ISPS_vs_HBPS). b. The heatmap of differential gene expression two comparisons (25ºC ISPS_vs_HBPS and 35ºC ISPS_vs_HBPS). The initiation stages of petals/petal-like structures and stamens/stamen-like structures (ISPS); the stage in which the hypanthium starts to sink below the perianth and stamens (HBPS).


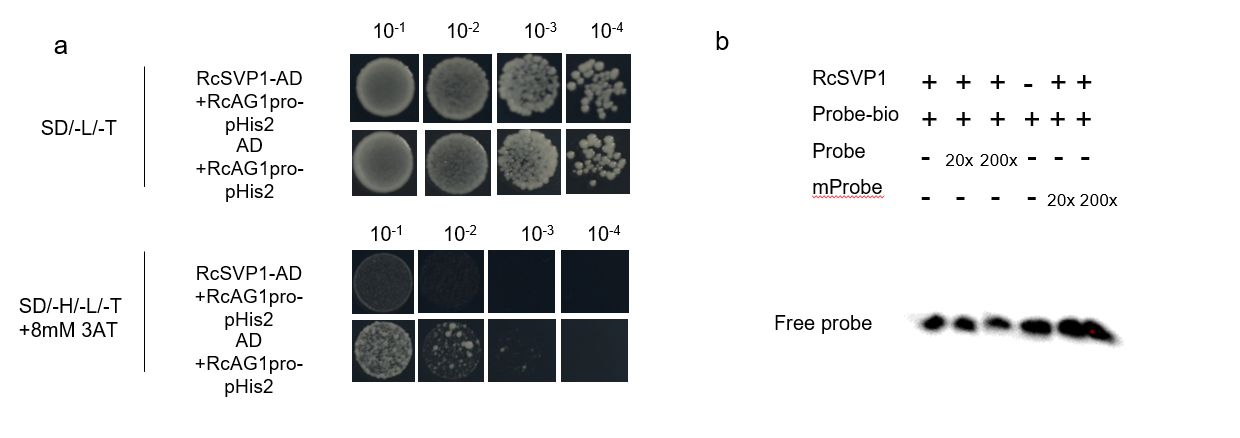


Fig. S24. Yeast one-hybrid (Y1H) assay and electrophoretic mobility shift assay (EMSA). a. Yeast one-hybrid (Y1H) assay of RcSVP1 and *RcAG1pro*. b. Electrophoretic mobility shift assay (EMSA) of RcSVP1 and *RcAG1pro*.


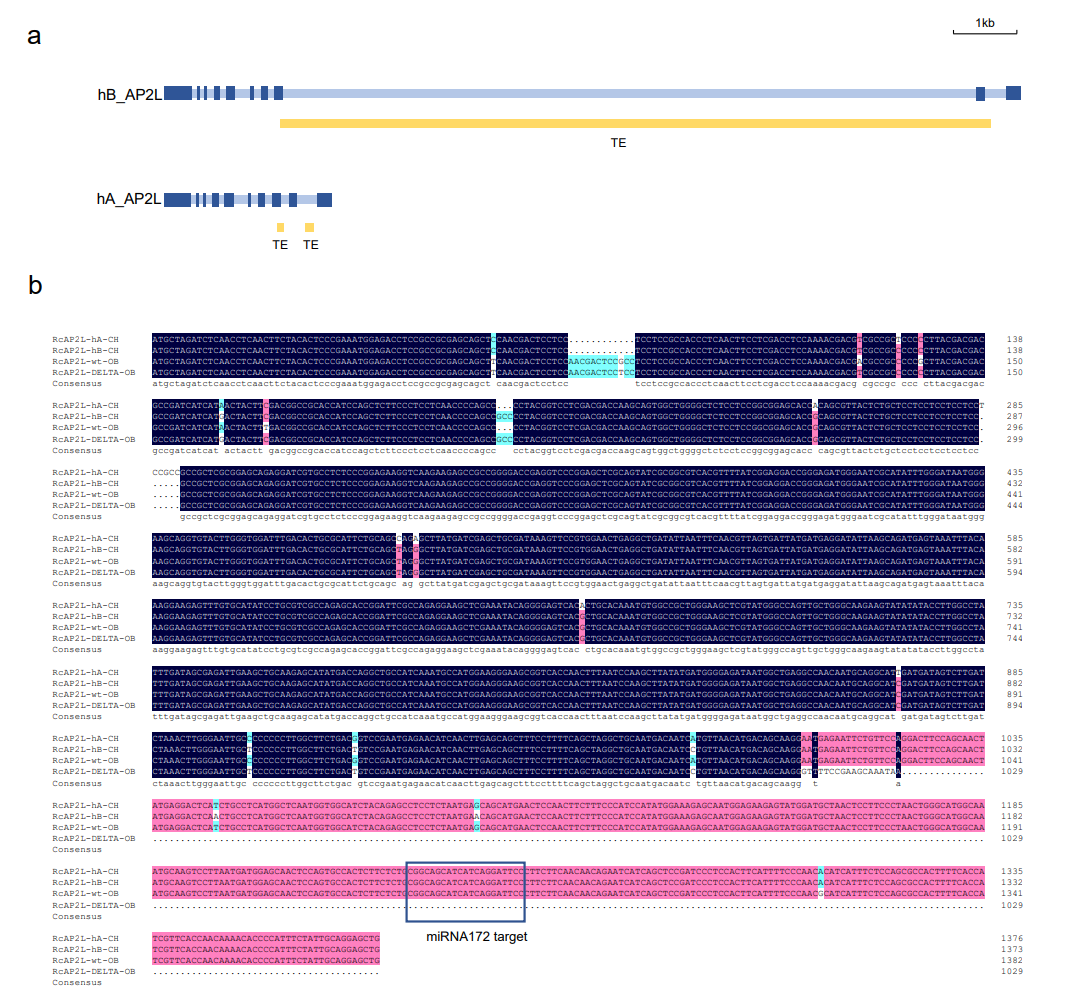


Fig. S25. Genotype information of *RcAP2L*. a. Two RcAP2L alleles of ‘CH’. Light blue, dark blue and yellow represent introns, exons, transposable element (TE), respectively. b. Sequence alignment of the CDS of *RcAP2L* in ‘OB’ and ‘CH’. The blue box is the binding site for miR172.


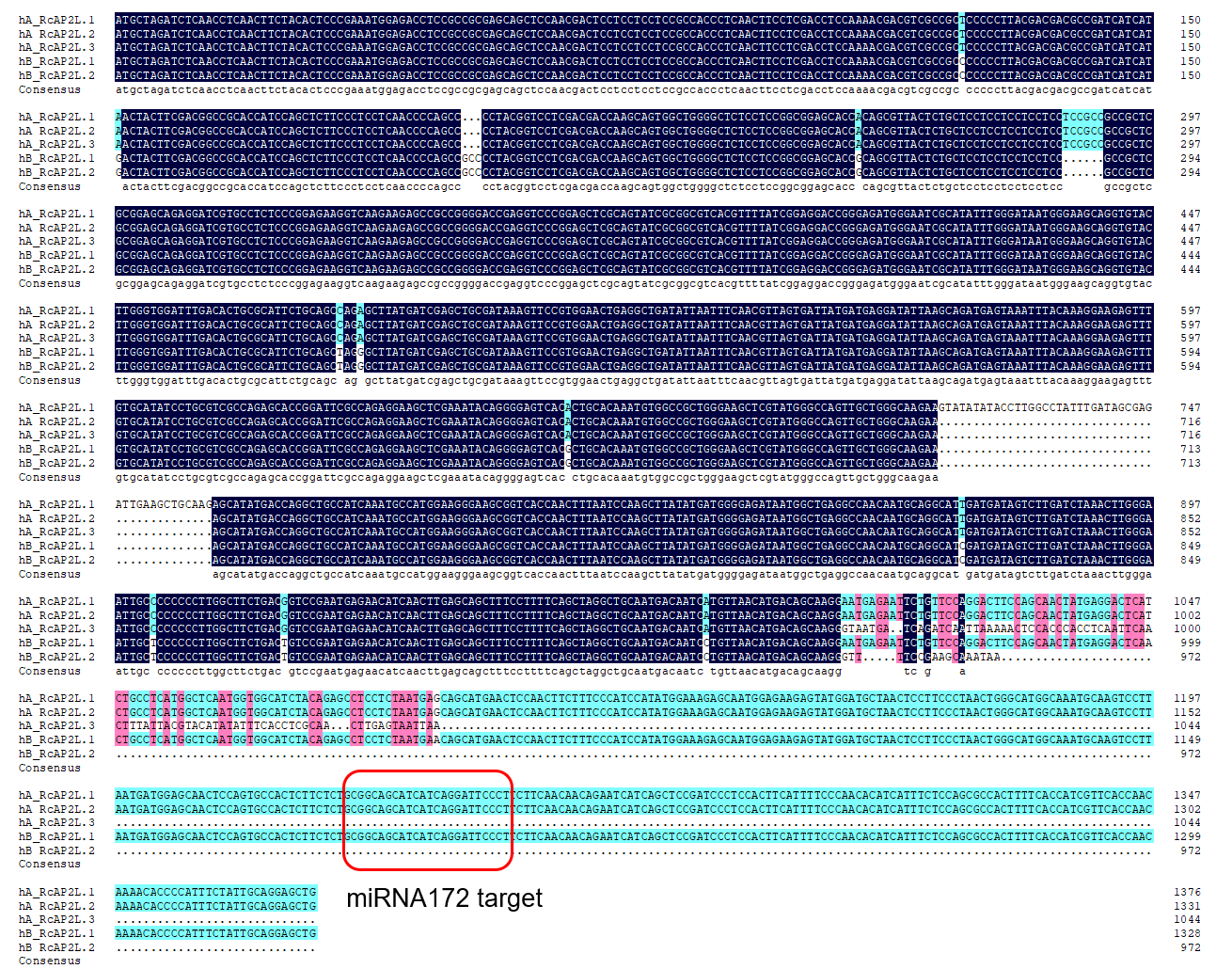


Fig. S26. *RcAP2L* variable spliceosome sequence alignment analysis. Boxed out in red are miRNA172 binding sites.
